# *Xanthoceras sorbifolium* Bunge Leaf Extract Ameliorates Diabetic Nephropathy Through Coordinated Metabolic Reprogramming and Inflammatory Signaling

**DOI:** 10.3390/foods15142576

**Published:** 2026-07-22

**Authors:** Mengting Han, Xianyu Zhang, Yiqing Jia, Yifei Zhang, Zijin Qin, Shuyu Zhou, Zhe Xu, Hui Zhou

**Affiliations:** 1 Key Laboratory of Biotechnology and Bioresources Utilization, Ministry of Education, Dalian Minzu University, Dalian 116600, China; 2 Department of Food Science and Technology, University of Georgia, Athens, GA 30602, USA

**Keywords:** *Xanthoceras sorbifolium* bunge leaf extract, diabetic nephropathy, flavonoids, metabolic reprogramming, inflammatory signaling

## Abstract

Diabetic nephropathy (DN) is a major complication of diabetes, yet effective dietary interventions remain limited. *Xanthoceras sorbifolium* Bunge leaf extract (XBL) has shown promising bioactivities, but its active components and mechanisms are not well understood. In this study, a comprehensive investigation including chemical profiling, network pharmacology, in vivo validation, and renal multi-omics was conducted. The results show a diverse composition dominated by flavonoids, which were identified as key bioactive contributors. In the streptozotocin-induced DN rat model, XBL administration significantly improved metabolic disorders and renal injury. Integrated metabolomic and transcriptomic analyses demonstrated that XBL alleviates DN by coordinately regulating metabolic reprogramming and inflammation-related signaling pathways. Additionally, representative flavonoids showed α-glucosidase inhibitory activity, supporting their potential role in glucose metabolic regulation. The results from this study suggest that XBL is a promising functional food ingredient for DN intervention.

## 1. Introduction

Diabetes mellitus (DM) is a metabolic disease marked by sustained hyperglycemia caused by defective insulin secretion and insulin resistance [1]. Data from the Lancet Global Burden of Disease showed that the diabetes population worldwide is expected to surpass 1.3 billion by 2050 [2], driven mainly by obesity, sedentary lifestyles, and population aging [3]. Chronic hyperglycemia disturbs glucose and lipid metabolism, leading to oxidative stress and chronic inflammation, and organ damage.

Progressive microvascular complications, such as diabetic nephropathy (DN), are common in patients. DN remains a leading cause of end-stage renal disease (ESRD) worldwide [4]. The pathogenesis of DN is mainly attributed to the thickening of the glomerular basement membrane and mesangial expansion, podocyte injury and hyaline arteriolosclerosis in the kidney, with clinical manifestations of persistent proteinuria, hypertension and progressive loss of renal function [5]. Currently, treatments mainly focus controlling blood glucose and blood pressure, including interventions targeting the renin–angiotensin–aldosterone system (RAAS) and antidiabetic medications such as glucagon like peptide 1 receptor agonists (GLP-1RAs) and sodium glucose cotransporter 2 (SGLT-2) inhibitors [6,7,8]. Although these treatments provide renal and cardiovascular benefits, their effects are limited by the restricted range of targeted pathways as well as the side effects induced by chronic treatment [9]. Increasing evidence indicates that DN is accompanied by profound renal metabolic reprogramming, which is closely intertwined with inflammation- and fibrosis-related signaling. Therefore, further understanding of the impact of metabolic alterations on transcriptional remodeling at the tissue level is essential for developing more effective intervention strategies against DN.

In recent years, plant-derived natural compounds have emerged as promising alter-natives for the management of metabolic and renal disorders due to their multi-component composition, multi-target regulatory potential, and favorable safety profiles [10]. *Xanthoceras sorbifolium* Bunge, commonly known as yellowhorn, is a deciduous shrub native to northern China with high ecological, economic, and medicinal value [11]. Phytochemical investigations have reported that its leaves contain abundant bioactive constituents, such as triterpenoids, flavonoids, phenolic acids, and saponins, which are associated with antioxidant, anti-inflammatory, and lipid-regulating effects [12]. In traditional use, the leaves of *Xanthoceras sorbifolium* Bunge have been prepared as an herbal tea to help regulate blood glucose, lipid levels, and uric acid, indicating that the leaf extract may have value as a functional food or nutraceutical ingredient [13]. Unlike the seeds, which are primarily exploited as oil-rich energy sources or biodiesel feedstocks, the leaves represent a distinct, underutilized agricultural by-product with greater potential for long-term metabolic intervention [14]. However, earlier investigations mainly focused on its seeds and oils [15], whereas the biological functions and health-promoting potential of the leaves remain insufficiently understood. Moreover, current research on leaf extracts has primarily focused on a single metabolic pathway and lacks integrated regulation of renal metabolism and inflammatory pathways [16]. Additionally, kidney-specific multi-omics investigations linking transcriptional changes with metabolic alterations remain scarce for *Xanthoceras sorbifolium* Bunge leaf extract (XBL), which may limit mechanistic understanding and hinder its commercialization as a functional food ingredient. Flavonoids are the major bioactive components in XBL and are well-documented for their regulatory effects on glucose metabolism. However, direct functional validation of these predicted core constituents remains limited. As amino acid metabolism, carbohydrate metabolism, and vitamin-associated pathways represent key targets of dietary intervention, elucidating the involvement of XBL in renal metabolic reprogramming has important scientific value and practical significance.

Therefore, this study aimed to systematically elucidate the pharmacological basis and multi-target synergistic mechanisms underlying the renoprotective effects of XBL against diabetic nephropathy through an integrated multi-omics strategy. Specifically, the chemical profile of XBL was characterized using ultra-performance liquid chromatography-tandem mass spectrometry (UPLC-MS/MS). Network pharmacology analysis was then conducted to select potential bioactive constituents, core targets, and DN-associated signaling pathways. These findings were subsequently validated through in vivo pharmacodynamic evaluation, together with renal metabolomic and transcriptomic profiling. Integrated transcriptome-metabolome association analysis, including gene-metabolite correlation, joint pathway enrichment, and network reconstruction, were then conducted to characterize the coordinated regulatory mechanisms underlying XBL-mediated renoprotection. Additionally, the α-glucosidase inhibitory activities of XBL and its major flavonoids were evaluated to provide targeted functional validation of the predicted core constituents. The results of this study provide pharmacological and multi-omics support for the potential use of XBL as a functional food ingredient for diabetic nephropathy intervention.

## 2. Materials and Methods

### 2.1. Materials and Reagents

The XBL aqueous extract was purchased from Ningxia Vanilla Biotechnology Co., Ltd. (Ningxia, China). According to the manufacturer, dried XBL leaves were extracted with distilled water at a solid-to-liquid ratio of 1:10 (*w*/*v*) under reflux for 1 h, and the extraction was repeated twice. The combined extracts were filtered, concentrated under reduced pressure, and spray-dried to obtain a powdered extract, which was stored at −20 °C until use. Acetonitrile and methanol (HPLC grade) were purchased from Thermo Fisher Scientific (Loughborough, UK), and formic acid was obtained from TCI Co., Ltd. (Shanghai, China). 2-Amino-3-(2-chlorophenyl)-propionic acid was obtained from Aladdin Biochemical Technology Co., Ltd. (Shanghai, China). Ammonium formate and streptozotocin (STZ) were obtained from Sigma (St. Louis, MO, USA). The blood glucose meter and corresponding test strips were purchased from Beijing Yicheng Bioelectronic Technology Co., Ltd. (Beijing, China). Commercial assay kits for total cholesterol (TC), triglycerides (TG), urine protein (PRO), serum creatinine (Scr), and blood urea nitrogen (BUN) were obtained from Nanjing Jiancheng Bioengineering Institute (Nanjing, China). α-Glucosidase from yeast (S10050, 100 U/mg) and p-nitrophenyl-α-D-glucopyranoside (p-NPG, S10137) were purchased from Shanghai Yuanye Biotechnology Co., Ltd. (Shanghai, China). Reference standards (purity > 98%), including kaempferol, luteolin, epicatechin, taxifolin and norwogonin, were purchased from Shanghai Winherb Medical Science Co., Ltd. (Shanghai, China). Sodium carbonate was purchased from Tianjin Kemiou Chemical Reagent Co., Ltd. (Tianjin, China). All other reagents and chemicals used in the study were of analytical grade, unless otherwise indicated.

### 2.2. UHPLC–Orbitrap HRMS/MS Analysis of XBL Extract

The XBL extract was analyzed using UHPLC coupled with Orbitrap high-resolution mass spectrometry (UHPLC–Orbitrap HRMS). Chromatographic separation was performed an ACQUITY UPLC I-Class (Waters Corporation, Milford, MA, USA) coupled with an ACQUITY UPLC HSS T3 column (100 mm × 2.1 mm, 1.8 μm; Waters Corporation, Milford, MA, USA). The injection volume was 2 μL. The column temperature was 45 °C and the solvent flow rate was 0.35 mL/min. Solvent A was 0.1% formic acid, and solvent B was acetonitrile. The gradient elution procedure was set as follows: 0–2 min, 5% B; 2–4 min, 5–30% B; 4–8 min, 30–50% B; 8–10 min, 50–80% B; and 10–14 min, 80–100% B.

The MS detection was performed using a Q Exactive HF Orbitrap (Thermo Fisher Scientific, San Jose, CA, USA) with an electrospray ionization (ESI) source. The spray voltage was 3.8 kV in positive mode and 3.2 kV in negative mode. The other parameters were set as follows: sheath gas, 35 arb. units; auxiliary gas, 8 arb. units; capillary temperature, 320 °C; auxiliary gas heater temperature, 350 °C; and S-Lens RF level, 50. Full MS acquisition was performed across an *m*/*z* range of 100–1500. Data-dependent MS/MS analysis was carried out using stepped normalized collision energies (NCEs) of 10, 20, and 40 eV. Metabolite annotation was performed by referencing mzCloud and the Human Metabolome Database (HMDB).

### 2.3. Network Pharmacology Analysis

Constituents identified by UPLC–MS/MS were then screened through the Traditional Chinese Medicine System Pharmacology Database (TCMSP), using oral bioavailability (OB ≥ 20%) and drug-likeness (DL ≥ 0.18) as selection criteria. Putative targets for these compounds were retrieved from SwissTargetPrediction, while DN-related targets were retrieved from GeneCards and OMIM using ‘diabetic nephropathy’ as the search keyword. All target names were then standardized using the UniProt database. The overlapping genes between compound-associated targets and disease-associated targets were illustrated by a Venn diagram and considered candidate therapeutic targets.

Protein–protein interaction (PPI) networks for *Homo sapiens* were constructed using the STRING database with a minimum confidence score of 0.4. The networks were imported into Cytoscape v3.9.1 for visualization and topological analysis. Hub genes were identified using the CytoNCA plugin based on betweenness centrality (BC), closeness centrality (CC), degree centrality (DC), and eigenvector centrality (EC). Gene Ontology (GO) functional annotation and Kyoto Encyclopedia of Genes and Genomes (KEGG) pathway enrichment analyses were performed using the DAVID database, with *p* < 0.05 considered statistically significant.

### 2.4. Experiment Design for Rat Study

Male Sprague-Dawley (SD) rats (180 ± 20 g; Liaoning Changsheng Biotechnology Co., Ltd., Benxi, China) were housed under controlled conditions at 23 ± 2 °C with a 12 h light/dark cycle and ad libitum access to food and water. All animal experiments were approved by the Scientific Ethics Committee of Dalian Minzu University (Approval No. 202502006).

Following a one-week adaptation period, a total of 24 rats were randomly allocated into three groups (*n* = 8 per group): the normal control group (Control), the diabetic nephropathy model group (Model), and the XBL-treated group (XBL). Rats in the Control group were fed a standard diet, whereas those in the Model and XBL groups were administered a high-fat and sucrose diet to induce insulin resistance. After eight weeks of dietary intervention, T2DM was induced by a single intraperitoneal injection of STZ (35 mg/kg). Rats with fasting blood glucose (FBG) levels ≥ 16.7 mmol/L one week after STZ injection were considered diabetic. Subsequently, diabetic rats were randomized into the Model and XBL groups using a minimization method based on FBG levels to ensure comparability at baseline. At the time of randomization, the mean FBG levels in the Model and XBL groups were 21.95 and 21.68 mmol/L, respectively, with an intergroup difference of 0.27 mmol/L (≤1 mmol/L, *p* = 0.885). During the following 8-week treatment period, rats in the XBL group received daily gavage of XBL extract at 2 g/kg, whereas rats in the Control and Model groups received an equal volume of normal saline.

### 2.5. Biochemical Analysis

During this 8-week treatment period, FBG levels were measured once weekly, and physiological and biochemical parameters were assessed using all eight biological rep-licates from each group. Briefly, overnight fasted rats were bled from the tail vein, and glucose levels were measured using a blood glucose meter. At the end of the experiment, serum and urine samples were collected for biochemical analysis. TC, TG, BUN, Scr, Ucr, and PRO levels were measured using the corresponding assay kits. Kidney weight (mg) and body weight (g) were recorded to calculate the kidney index according to the equation below.

### 2.6. Histopathological Analysis

Kidney and pancreas tissues were collected after 8 weeks of treatment for histopathological analysis. The samples (*n* = 3) were fixed in 4% paraformaldehyde for 24 h, dehydrated in a graded ethanol series, washed in xylene, and embedded in paraffin. Paraffin blocks were sectioned, deparaffinized, rehydrated, and stained with hematoxylin and eosin (H&E). Histopathological evaluation was performed in a blinded manner by an experienced pathologist who was unaware of the experimental group assignments and sample identifiers throughout the assessment. Histopathological changes in the glomeruli, renal tubules, and pancreatic islets were observed and photographed under a light microscope.

### 2.7. UPLC–MS/MS-Based Renal Metabolomics

**Sample preparation:** For renal metabolomic profiling, a subset of six biological replicates (*n* = 6) was randomly selected from each of the three experimental groups. Kidney tissues were harvested and immediately snap-frozen in liquid nitrogen for subsequent extraction and analysis. Prior to processing, frozen renal tissues were thawed and homogenized on ice. The homogenates were then mixed with 400 μL of methanol, and vortex mixed and centrifuged at 12,000 rpm for 10 min at 4 °C. The resulting supernatant was dried in a vacuum. The residue was dissolved in 150 μL of 80% methanol with 2-chloro-L-phenylalanine. The extract was then filtered and transferred to LC–MS vials for analysis.

**Chromatographic parameters:** Sample analysis was carried out on a Vanquish UPLC system, using the above-described ACQUITY UPLC HSS T3 C18 column. The column temperature was maintained at 40 °C, the flow rate was set to 0.3 mL/min and an injection volume of 2 μL was applied. Under positive ionization conditions, mobile phase A was water containing 0.1% formic acid, and mobile phase B was acetonitrile. Under negative ionization conditions, 5 mM ammonium formate aqueous solution was used as mobile phase A, whereas pure acetonitrile served as mobile phase B. The elution gradient was as follows: 0–1 min, 10% B; 1–5 min, 10–98% B; 5–6.5 min, 98% B; 6.5–6.6 min, 98–10% B; and 6.6–8 min, 10% B.

**Mass spectrometry conditions:** Detection was performed using an MS equipped with an ESI source. MS full scans and MS/MS spectra were collected simultaneously. The instrument parameters were set as follows: sheath gas flow, 40 arb; auxiliary gas flow, 10 arb; spray voltage, +3.50 kV in positive mode and −2.50 kV in negative mode; and capillary temperature, 325 °C. MS^1^ spectra were acquired over an *m*/*z* range of 100–1000 at a resolving power of 60,000 FWHM. MS^2^ spectra were acquired at a resolving power of 15,000 FWHM. Data-dependent acquisition (DDA) was performed with four scans per cycle, a collision energy of 30%, and dynamic exclusion.

**Data processing and metabolite identification:** MS data were converted to mzXML format by MSConvert (ProteoWizard v3.0.8789, Palo Alto, CA, USA). Next, peak picking, retention time shift and alignment of chromatograms were performed for the converted files using XCMS v3.12.0 (La Jolla, CA, USA) in the R (R Foundation for Statistical Computing, Vienna, Austria). Peak areas were normalized using internal standard. The metabolites were identified by comparing the exact fragments mass of the unknown metabolites to databases, including HMDB, MassBank, KEGG, LipidMaps, mzCloud and an internal library from Panomix Biomedical Tech Co., Ltd. (Suzhou, China). The complete raw metabolomics dataset is available in Supplementary Table S4.

### 2.8. Renal Transcriptomic Analysis

Renal transcriptomic analysis was performed on a subset of four biological replicates (*n* = 4) per group. Total RNA was isolated from kidney tissues using TRIzol reagent (Invitrogen, Carlsbad, CA, USA). RNA concentration and purity were measured using a NanoDrop 2000 spectrophotometer (Thermo Fisher Scientific, Waltham, MA, USA), and RNA integrity was assessed using an Agilent 2100 Bioanalyzer (Agilent Technologies, Santa Clara, CA, USA). Only samples with an RNA integrity number (RIN) ≥ 7.0 were used for library preparation.

The VAHTS Universal V6 RNA-seq Library Prep Kit (Vazyme, Nanjing, China) was used for library preparation. Paired end sequencing was performed on an Illumina platform by OE Biotech Co., Ltd. (Shanghai, China). After quality control, clean reads were mapped to the rat reference genome. Gene expression levels were calculated as fragments per kilobase of exon per million mapped reads (FPKM). Differentially expressed genes (DEGs) were identified using DESeq2 v1.20.0, with |fold change| ≥ 2 and adjusted *p* < 0.05 as the screening criteria. GO and KEGG enrichment analyses were then performed to evaluate the biological functions and pathways associated with the DEGs.

### 2.9. Metabolomic and Transcriptomic Joint Analysis

A combined metabolomic and transcriptomic analysis was performed to investigate the association between the changes in gene expression in the kidney and metabolic changes. Pearson correlation coefficients between DEGs and differentially expressed metabolites (DEMs) were calculated and correlation heatmaps were generated. Correlations with *p* < 0.05 and |*r*| > 0.6 were considered significant, and the correlation network was constructed in Cytoscape. Joint pathway analysis of DEGs and DEMs was performed using the MetaboAnalyst platform. Pathway enrichment analysis was conducted using hypergeometric testing with the “All pathways (integrated)” option and pathway topology was calculated using degree centrality with the “Combine queries” integration option.

Additionally, the Network Explorer module based on the KEGG Global Metabolic Network was used to construct integrated gene–metabolite–pathway networks, which were visualized using Kyoto Encyclopedia of Genes and Genomes Markup Language (KGML) files.

### 2.10. Efficiency Validation of Selected Flavonoids

The representative flavonoids were subjected to α-glucosidase-related assays, including inhibitory activity determination, enzyme kinetic analysis, circular dichroism spectroscopy, and molecular docking. Detailed experimental procedures are provided in the Supplementary Materials (Methods S1–S4).

### 2.11. Statistical Analysis

Data are presented as the mean ± standard deviation (SD). Statistical analysis was conducted using GraphPad Prism 9.0 (GraphPad Software, San Diego, CA, USA). Multiple group comparisons were performed by one-way analysis of variance (ANOVA) with Tukey’s post hoc test. The *p* value < 0.05 was considered as statistically significant.

## 3. Results

### 3.1. Chemical Profiling and Network Pharmacology Analysis

#### 3.1.1. Chemical Composition of XBL

The Total ion chromatograms (TICs) profiles of the XBL extract are shown in Figure S1. In total, 293 chemical components were putatively assigned (Table S1), indicating its chemical complexity. These constituents were mainly classified as flavonoids, terpenoids, phenolic acids, phenylpropanoids, and other secondary metabolites, with flavonoids and terpenoids as the predominant groups.

To understand the potential mechanisms of action, representative constituents were selected based on ADME properties. Among them, 18 constituents with high oral bioavailability and drug likeness were selected for further network pharmacology analysis.

#### 3.1.2. Network Pharmacology Analysis of Bioactive Compounds and Potential Targets

**Figure 1:**
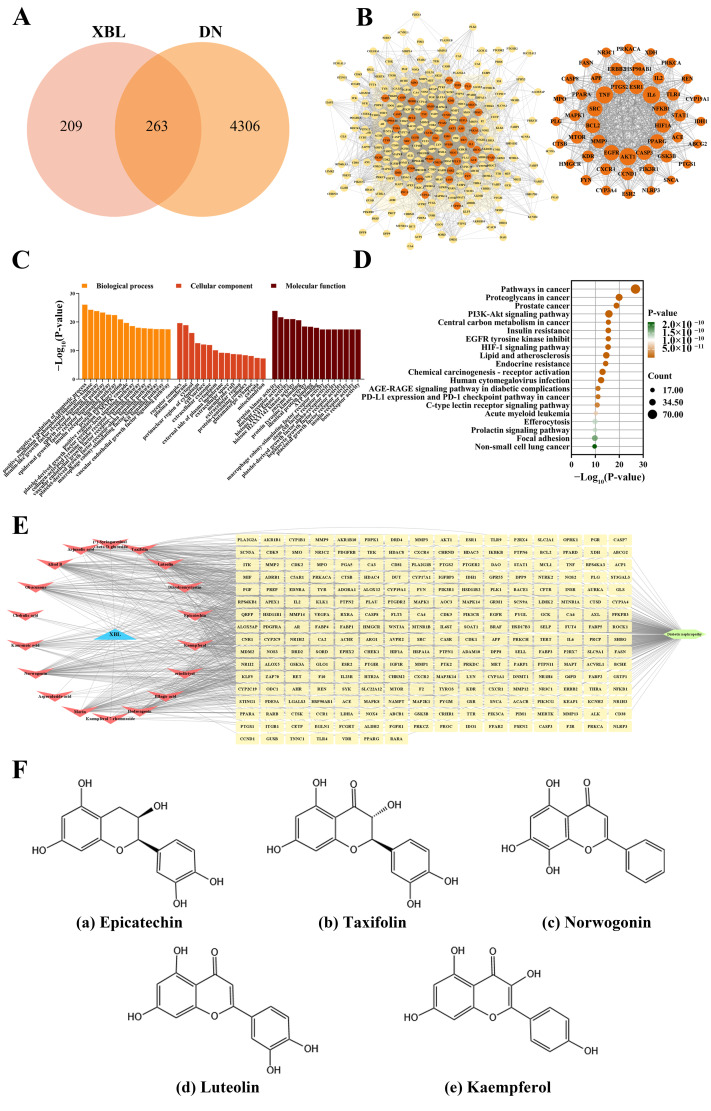
Network pharmacology analysis of XBL. (**A**) Venn diagram showing the overlapping targets between XBL compounds and diabetic nephropathy-related genes. (**B**) Protein–protein interaction (PPI) network of the intersecting targets. (**C**) Gene Ontology (GO) enrichment analysis of the intersecting targets, including biological process (BP), cellular component (CC), and molecular function (MF). (**D**) KEGG pathway enrichment analysis of the overlapping targets. Bubble size and color reflect gene count and statistical significance, respectively. (**E**) Compound-target-disease network constructed based on XBL active ingredients, their predicted targets, and disease targets. (**F**) Topological analysis of XBL components within the composite target network based on Degree Centrality, identifying and ranking the top five components. (**a**) Epicatechin, (**b**) Taxifolin, (**c**) Norwogonin, (**d**) Luteolin, (**e**) Kaempferol.

To investigate the molecular mechanisms of XBL against DN, network pharmacology analysis was conducted. A total of 4569 DN related targets were retrieved from GeneCards and OMIM, and 472 potential targets of the screened XBL constituents were obtained from SwissTargetPrediction. Intersection analysis identified 263 shared targets between XBL and DN (Figure 1A). The PPI network constructed from these targets contained 261 nodes and 4521 edges (Figure 1B). 

GO and KEGG enrichment analyses showed that the shared targets were mainly associated with PI3K-Akt, HIF-1, and AGE-RAGE signaling pathways in diabetic complications, as well as biological processes related to receptor complex organization, plasma membrane localization, protein phosphorylation, and kinase activity regulation (Figure 1C,D). Network topology analysis identified IL6, AKT1, TNF, and EGFR as highly connected nodes in the PPI network. The results of compound-target-disease network indicating associations among potential XBL components and DN-associated targets are shown in Figure 1E. Five flavonoids were identified as core candidate compounds in the compound–target network, including epicatechin, taxifolin, norwogonin, luteolin, and kaempferol (Figure 1F and Table 1).

Collectively, the results of network pharmacology analysis identified candidate compounds, shared targets, and related signaling cascades, thereby establishing a foundation for the subsequent in vivo pharmacodynamic assessment and kidney metabolomic and transcriptomic profiling.

### 3.2. Therapeutic Efficacy of XBL Against Metabolic and Histological Abnormalities in DN Rats

**Figure 2:**
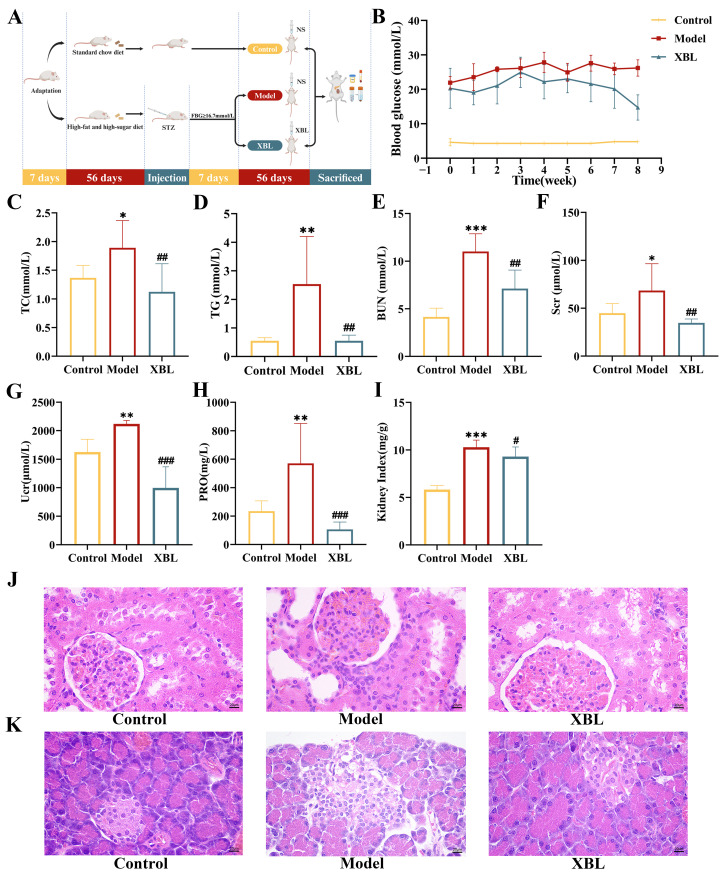
Experimental design and therapeutic effects of XBL on STZ-induced diabetic nephropathy (DN) in rats. (**A**) Schematic illustrating the experimental workflow comprising diet induction, STZ injection, and XBL treatment. (**B**) Weekly changes in fasting blood glucose levels during the 8-week intervention. (**C**–**F**) Serum levels of TG, TC, BUN, and Scr. (**G**,**H**) Urinary levels of Ucr and PRO. (**I**) Kidney index in each group. (**J**) Representative H&E staining of kidney tissues showing glomerular and tubular morphology. (**K**) H&E staining of pancreatic islet architecture after the 8-week intervention. Data are presented as mean ± SD. * *p* < 0.05, ** *p* < 0.01, *** *p* < 0.001 vs. Control group; # *p* < 0.05, ## *p* < 0.01, ### *p* < 0.001 vs. Model group.

To evaluate the therapeutic effect of XBL on DN, a rat model of type 2 diabetes was induced by a high-fat/high-sucrose diet combined with STZ injection. Once the model was successfully established, FBG in diabetic rats rose sharply to more than 16.7 mmol/L and remained significantly higher compared with the control group. The experimental design is shown in Figure 2A. After eight weeks of XBL intervention, FBG was greatly decreased compared with the untreated model group (Figure 2B).

Serum lipid profiling indicated disordered lipid metabolism in diabetic rats. Compared with the control group, the model group showed significantly increased TC and TG levels. XBL treatment reduced both TC and TG levels compared with the model group (Figure 2C,D). 

Renal function indicators were also altered in diabetic rats. Compared with the control group, the model group showed higher BUN and Scr levels, whereas XBL treatment reduced both levels after 8 weeks (Figure 2E,F). Urinary biochemical indices further reflected renal impairment in diabetic rats, as evidenced by increased Ucr and PRO levels. XBL administration significantly reduced Ucr and PRO levels compared with the model group (Figure 2G,H). In addition, the kidney index was significantly higher in diabetic rats than in controls, whereas XBL administration significantly reduced this index (Figure 2I).

Pathological analysis was consistent with the biochemical results. Kidney samples in the control group showed normal glomerular structure and well-maintained tubular architecture. However, samples from diabetic rats showed enlarged glomeruli, capillary dilation, and vacuolation and degeneration of renal tubular epithelial cells. These pathological changes were attenuated by XBL treatment (Figure 2J). For pancreatic tissues, compared with the control group, diabetic rats showed altered islet structure and irregular cell arrangement, whereas XBL-treated rats showed improved islet structure and more regular endocrine cell arrangement compared with the model group (Figure 2K).

### 3.3. Metabolic Changes in the Kidneys of DN Rats and Effects of XBL

**Figure 3:**
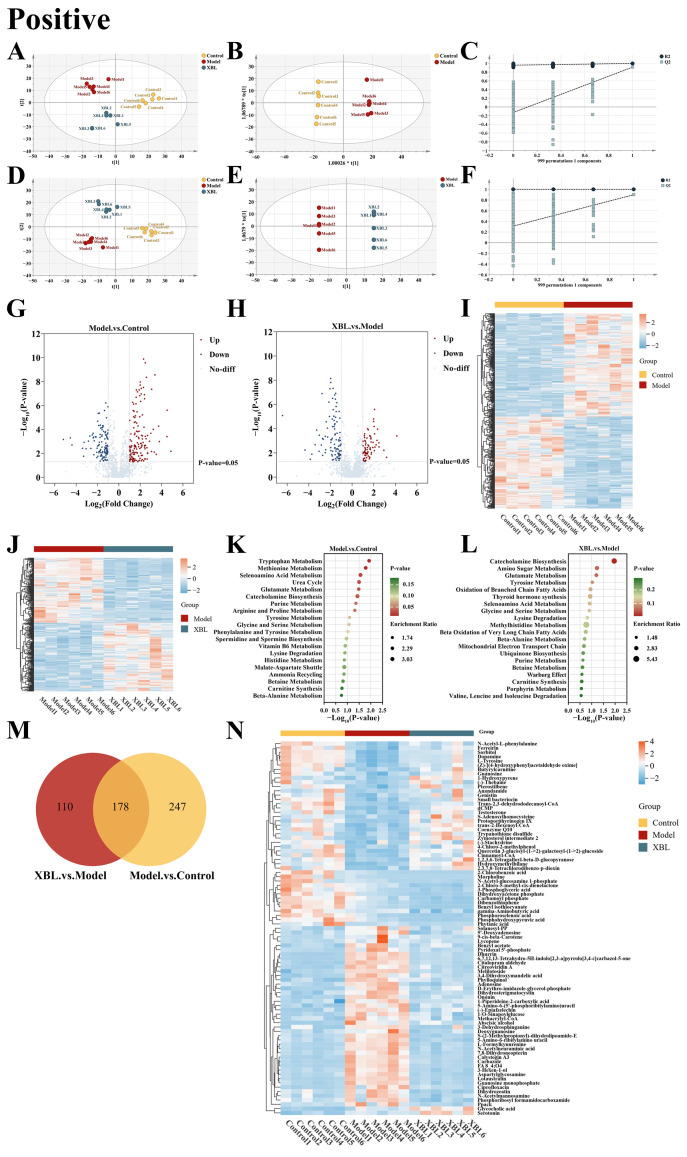
Metabolomic profiling of rat kidney tissues in positive ion mode. (**A**) Principal component analysis (PCA) score plot showing separation among Control, Model, and XBL groups (*n* = 6 per group). (**B**) Orthogonal partial least squares discriminant analysis (OPLS-DA) score plot of Model vs. Control. (**C**) Permutation validation plot for the OPLS-DA model of Model vs. Control (999 permutations). (**D**) Partial least squares discriminant analysis (PLS-DA) score plot showing separation among Control, Model, and XBL groups. (**E**) OPLS-DA score plot of XBL vs. Model. (**F**) Permutation validation plot for the OPLS-DA model of XBL vs. Model (999 permutations). (**G**,**H**) Volcano plots of significantly altered metabolites between Model vs. Control and XBL vs. Model. (**I**,**J**) Heatmaps showing the clustering of differential metabolites in Model vs. Control and XBL vs. Model. (**K**,**L**) KEGG pathway enrichment analyses of differential metabolites in Model vs. Control and XBL vs. Model, bubble size indicates the enrichment ratio, and color gradient reflects the *p*-value. (**M**) Venn diagram of overlapping differential metabolites between Model vs. Control and XBL vs. Model. (**N**) Heatmap of 178 overlapping differential metabolites identified in both comparisons.

**Figure 4:**
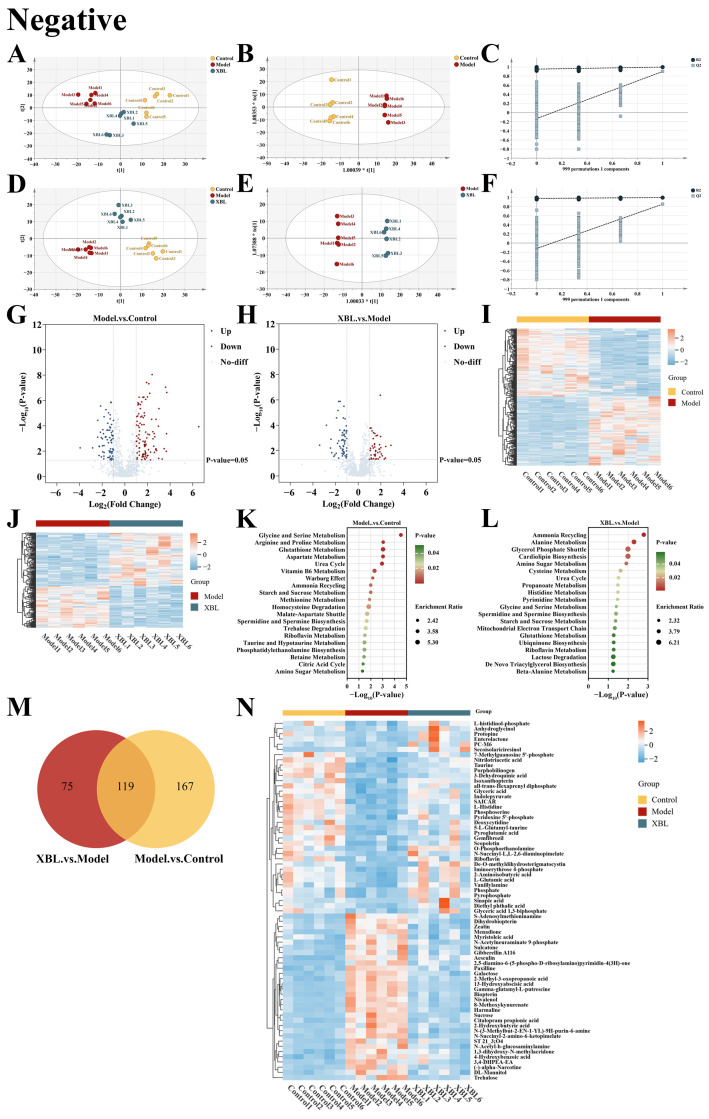
Metabolomic profiling of rat kidney tissues in negative ion mode. (**A**) Principal component analysis (PCA) score plot showing separation among Control, Model, and XBL groups (*n* = 6 per group). (**B**) Orthogonal partial least squares discriminant analysis (OPLS-DA) score plot of Model vs. Control. (**C**) Permutation validation plot for the OPLS-DA model of Model vs. Control (999 permutations). (**D**) Partial least squares discriminant analysis (PLS-DA) score plot showing separation among Control, Model, and XBL groups. (**E**) OPLS-DA score plot of XBL vs. Model. (**F**) Permutation validation plot for the OPLS-DA model of XBL vs. Model (999 permutations). (**G**,**H**) Volcano plots of significantly altered metabolites between Model vs. Control and XBL vs. Model. (**I**,**J**) Heatmaps showing the clustering of differential metabolites in Model vs. Control and XBL vs. Model. (**K**,**L**) KEGG pathway enrichment analyses of differential metabolites in Model vs. Control and XBL vs. Model, bubble size indicates the enrichment ratio, and color gradient reflects the *p*-value. (**M**) Venn diagram of overlapping differential metabolites between Model vs. Control and XBL vs. Model. (**N**) Heatmap of 119 overlapping differential metabolites identified in both comparisons.

Principal component analysis (PCA), partial least squares discriminant analysis (PLS-DA), and orthogonal partial least squares discriminant analysis (OPLS-DA) showed distinct dissimilarity among the Control, Model, and XBL groups in both positive and negative ion modes (Figure 3A–F and Figure 4A–F), indicating substantial changes in renal metabolism.

In positive ion mode, volcano plots and hierarchical clustering showed clear separation of metabolite profiles among the Control, Model, and XBL groups (Figure 3G–J). A total of 425 differential metabolites were identified in the Model group compared with the Control group, and 288 differential metabolites were identified in the XBL group compared with the Model group. Pathway analysis showed that these metabolites were mainly involved in amino acid metabolism, (e.g., tryptophan, methionine, glutamate, arginine, and proline), as well as nitrogen and purine metabolism (Figure 3K,L). Venn analysis identified 178 differential metabolites shared by both comparisons, and their relative abundance is shown in Figure 3M,N.

In negative ion mode, a total of 286 differential metabolites were identified in the Model group compared with the Control group, and 194 differential metabolites were identified in the XBL group compared with the Model group. Consistent with the positive ion mode results, volcano plots and hierarchical clustering showed clear metabolic separation among the groups (Figure 4G–J). Enrichment analysis showed that these metabolites were mainly associated with glycine, serine, arginine, and proline metabolism, glutathione metabolism, nitrogen metabolism, the urea cycle, and vitamin B6 metabolism (Figure 4K,L). Venn analysis identified 119 differential metabolites shared by both comparisons (Figure 4M,N).

**Figure 5:**
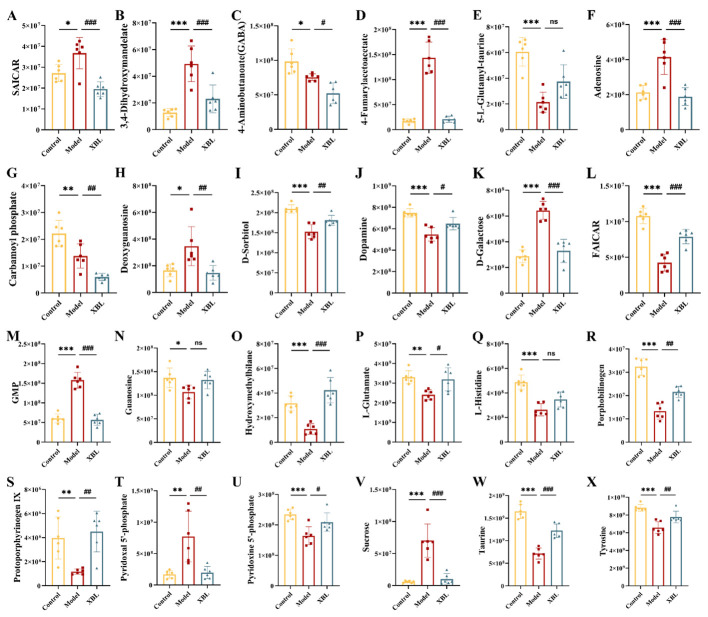
Relative abundance of representative differential metabolites among Control, Model, and XBL groups (*n* = 6 per group). (**A**) SAICAR. (**B**) 3,4-Dihydroxymandelate. (**C**) 4-Aminobutanoate (GABA). (**D**) 4-Fumarylacetoacetate. (**E**) 5-L-Glutamyl-taurine. (**F**) Adenosine. (**G**) Carbamoyl phosphate. (**H**) Deoxyguanosine. (**I**) D-Sorbitol. (**J**) Dopamine. (**K**) D-Galactose. (**L**) FAICAR. (**M**) GMP. (**N**) Guanosine. (**O**) Hydroxymethylbilane. (**P**) L-Glutamate. (**Q**) L-Histidine. (**R**) Porphobilinogen. (**S**) Protoporphyrinogen IX. (**T**) Pyridoxal 5′-phosphate. (**U**) Pyridoxine 5′-phosphate. (**V**) Sucrose. (**W**) Taurine. (**X**) Tyrosine. Data are presented as mean ± SD. * *p* < 0.05, ** *p* < 0.01, *** *p* < 0.001 vs. Control group; # *p* < 0.05, ## *p* < 0.01, ### *p* < 0.001 vs. Model group.

By integrating the differential metabolite profiles from the two ionization modes, 24 XBL responsive metabolites were screened (Table 2). These metabolites were associated with amino acid metabolic pathways (e.g., L-glutamate, tyrosine, taurine, dopamine), carbohydrate metabolism (e.g., sucrose, d-galactose, d-sorbitol), nucleotide metabolism (e.g., GMP, adenosine, deoxyguanosine), porphyrin metabolism (e.g., porphobilinogen, protoporphyrinogen IX, hydroxymethylbilane), and vitamin-related and intermediary metabolism (e.g., pyridoxine 5′-phosphate and pyridoxal 5′-phosphate) (Figure 5).

**Figure 6:**
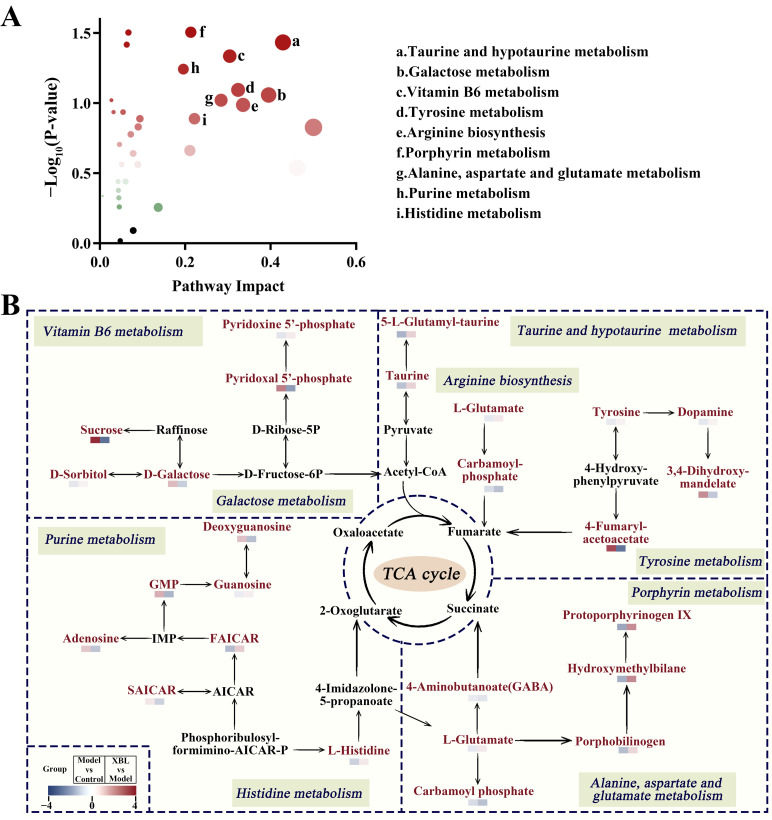
Overview of metabolic pathway alterations in DN rats after XBL intervention. (**A**) Metabolic pathway enrichment analysis based on identified differential metabolites, performed using MetaboAnalyst. The x-axis represents pathway impact, while the y-axis indicates the statistical significance of enrichment (−log_10_ *p*-value). (**B**) Network map of key metabolic pathways constructed based on identified differential metabolites from both Model vs. Control and XBL vs. Model comparisons. The diagram illustrates metabolic interconnections among galactose, purine, porphyrin, tyrosine, vitamin B6, taurine and hypotaurine, arginine, histidine, and glutamate-related pathways.

After XBL treatment, substantial enrichment was found in pathways such as taurine and hypotaurine metabolism, galactose metabolism, vitamin B6 metabolism, tyrosine metabolism, arginine biosynthesis, porphyrin metabolism, alanine, aspartate and glutamate metabolism, purine metabolism, and histidine metabolism (Figure 6A). Network based pathway mapping showed that these changes were connected within amino acid, carbohydrate, and nucleotide metabolism, indicating that XBL modulated coordinated renal metabolic alterations (Figure 6B).

### 3.4. Impact of XBL on the Renal Transcriptome in DN Rats

**Figure 7:**
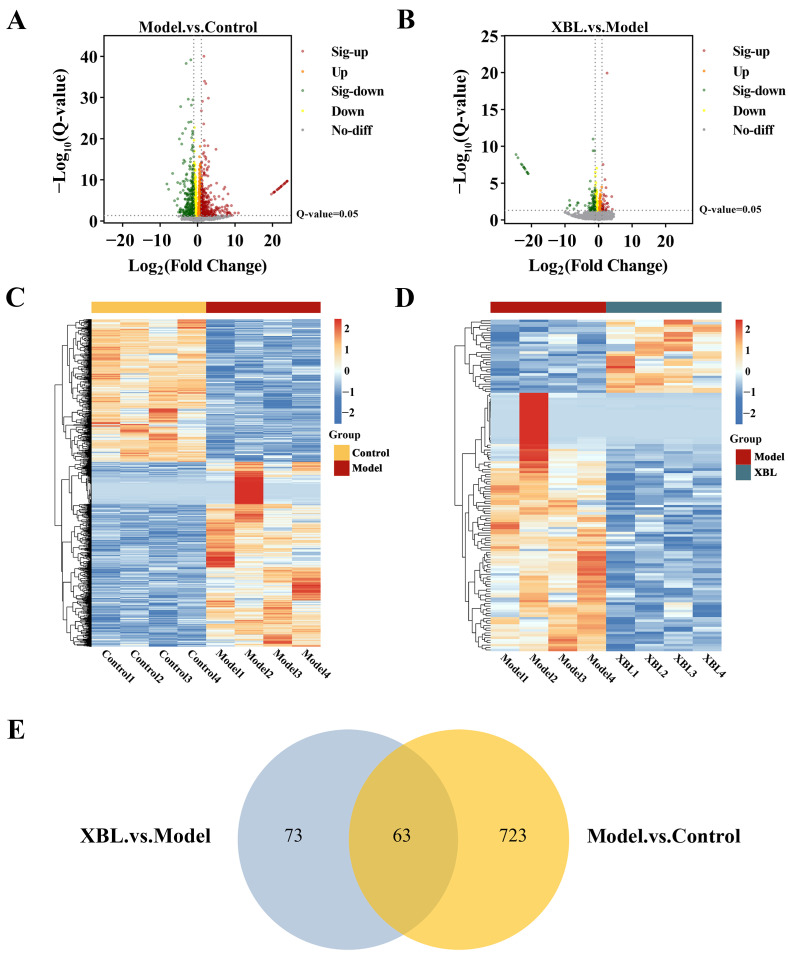
Transcriptomic analysis of differentially expressed genes (DEGs) in rat kidney tissues. (**A**,**B**) Volcano plots showing DEGs in the comparisons of Model vs. Control and XBL vs. Model. (**C**,**D**) Hierarchical clustering heatmaps of DEGs in Model vs. Control and XBL vs. Model. (**E**) Venn diagram showing overlapping DEGs between the Model vs. Control and XBL vs. Model groups.

Differential gene expression analysis is shown in Figure 7A,B. Compared with the Control group, 786 DEGs were identified in the Model group, including 444 upregulated and 342 downregulated genes. Compared with the Model group, 136 DEGs were identified in the XBL group, including 30 upregulated and 106 downregulated genes.

Hierarchical clustering also suggested different transcriptomic patterns among the Control, Model and XBL groups (Figure 7C,D). The Model group showed a disrupted transcriptional profile compared with the Control, whereas the XBL group showed partial recovery, with several transcripts exhibiting expression trends similar to those in the Control. These shared DEGs were subsequently used for enrichment analysis to investigate the biological functions and related signaling pathways. 

### 3.5. Functional Enrichment Analysis of Renal DEGs

**Figure 8:**
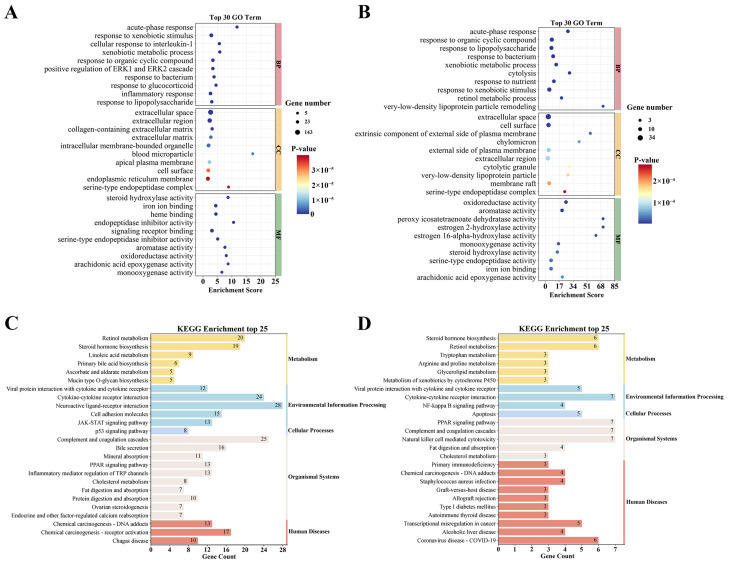
GO and KEGG pathway enrichment analysis of DEGs. (**A**,**B**) GO enrichment analysis of DEGs in Model vs. Control and XBL vs. Model, including biological process, cellular component, and molecular function categories. (**C**,**D**) KEGG pathway enrichment analysis of DEGs in Model vs. Control and XBL vs. Model. Gene counts and *p*-values are indicated for each enriched term or pathway.

To understand the biological impact of renal transcriptomic changes, the identified DEGs were analyzed using GO and KEGG enrichment approaches. Compared with the control, the DEGs from the Model group were predominantly involved in pathways of glucocorticoid response, inflammatory response, and lipopolysaccharide response. In the cellular component category, these genes were largely distributed in the extracellular region, cell surface, extracellular matrix, and endoplasmic reticulum membrane. GO molecular function enrichment mainly involved steroid hydroxylase and oxidoreductase activity, as well as heme and signaling receptor binding (Figure 8A).

Compared with the Model group, DEGs in the XBL group were mainly enriched in acute phase response, response to lipopolysaccharide, and retinol metabolic process. GO cellular component enrichment included extracellular space, cell surface, extracellular region, and very-low-density lipoprotein particle, whereas GO molecular function enrichment mainly involved steroid hydroxylase activity, oxidoreductase activity, and arachidonic acid epoxygenase activity (Figure 8B).

KEGG pathway analysis showed that DEGs identified between the Model and Control groups were mainly enriched in steroid hormone biosynthesis, cytokine-cytokine receptor interaction, PPAR signaling, JAK-STAT signaling, and inflammatory mediator regulation of TRP channels (Figure 8C). DEGs identified after XBL treatment were mainly enriched in PPAR signaling, cholesterol metabolism, NF-κB signaling, and type I diabetes mellitus pathways (Figure 8D).

**Figure 9:**
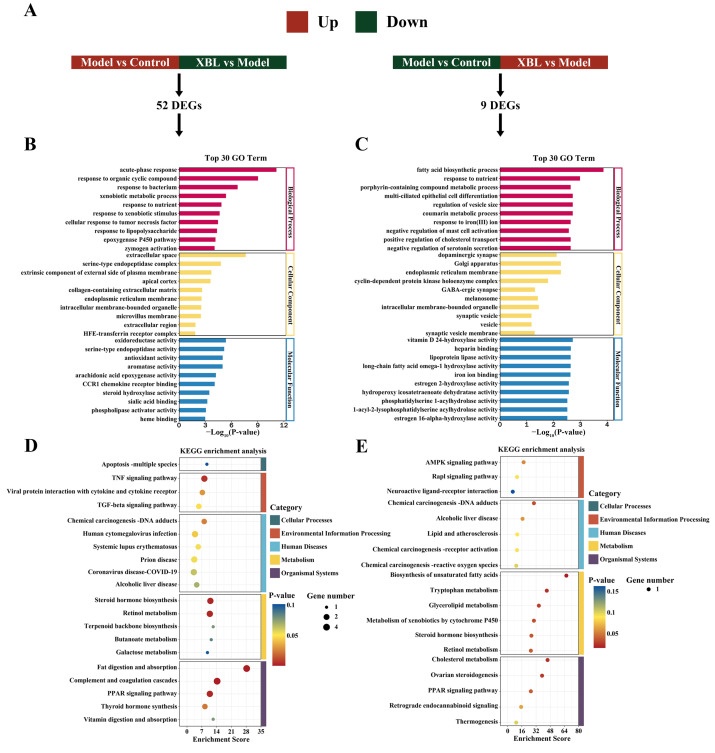
Functional enrichment analysis of DEGs reversed by XBL treatment in diabetic rats. (**A**) Venn diagrams showing DEGs with opposite expression trends in Model vs. Control and XBL vs. Model comparisons. (**B**,**C**) GO enrichment analysis of DEGs up-regulated in the Model group but down-regulated after XBL treatment, and DEGs down-regulated in the Model group but up-regulated after XBL treatment. (**D**,**E**) KEGG pathway enrichment analysis of the same two DEG subsets: up in Model and down after XBL treatment; down in Model and up after XBL treatment. Color scales indicate statistical significance (*p*-value), and dot size represents the number of genes enriched in each term.

To further examine the expression patterns of overlapping DEGs, these genes were grouped by direction of change. Among them, 52 genes were upregulated in the Model group and downregulated after XBL treatment, whereas 9 genes were downregulated in the Model group and upregulated after XBL treatment (Figure 9A). GO analysis showed that genes upregulated in the Model group but downregulated after XBL treatment were enriched in biological processes such as acute-phase response and response to organic cyclic compounds. These genes were also enriched in the extracellular space and serine-type endopeptidase complex (Figure 9B). However, genes downregulated in the Model group but upregulated after XBL treatment were enriched in fatty acid biosynthetic process, response to nutrient, and dopaminergic synapse. These genes were also associated with the Golgi apparatus (Figure 9C).

KEGG enrichment analysis showed that genes upregulated in the Model group and downregulated after XBL administration were mainly enriched in the TNF signaling pathway, steroid hormone biosynthesis, PPAR signaling pathway, retinol metabolism, complement and coagulation cascades, and TGF-β signaling pathway (Figure 9D). However, genes downregulated in the Model group but upregulated after XBL treatment were enriched in unsaturated fatty acid biosynthesis, cholesterol, glycerolipid, and tryptophan metabolism (Figure 9E).

To visualize expression changes in representative DEGs within selected signaling pathways, pathway maps of the PPAR, TGF-β, and TNF signaling pathways were generated from transcriptomic data (Figure S2). 

### 3.6. Integrated Transcriptomic and Metabolomic Analysis

Integrated analysis showed close association between renal transcriptional and metabolic alterations after XBL treatment in DN rats. Among the 24 DEMs and 61 DEGs, a substantial proportion of gene-metabolite pairs showed correlations (Table S3).

**Figure 10:**
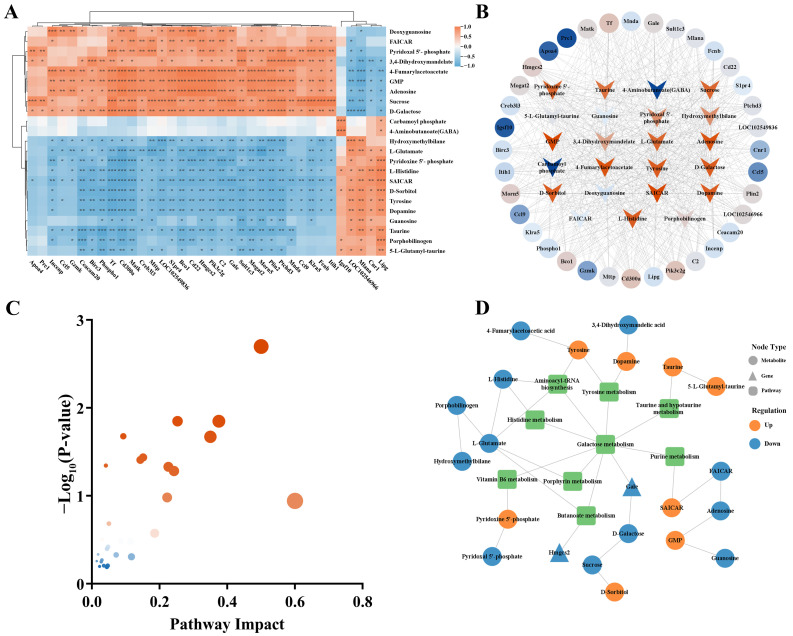
Integrated correlation analysis of renal transcriptomics and metabolomics. (**A**) Pearson correlation heatmap between DEGs and DEMs (*, ** and ***). (**B**) Correlation network of DEGs and DEMs constructed based on significant correlations (*p* < 0.05 and |*r*| > 0.6). (**C**) Integrated pathway analysis of transcriptomic and metabolomic alterations, with pathway impact indicating topological importance and −log10 (*p*-value) representing statistical significance. (**D**) KGML-based network illustrating the integrated relationships among DEGs, DEMs, and associated metabolic pathways.

As shown in Figure 10A, significant associations were observed between 23 DEMs and 36 DEGs (*p* < 0.05 and |*r*| > 0.6). The DEG-metabolite correlation network also indicated these associations (Figure 10B). *Prc1*, *Igsf10*, SAICAR, and D-galactose showed relatively high connectivity within the network.

Joint pathway analysis identified nine significant pathways, including the metabolism of galactose, tyrosine, taurine and hypotaurine, porphyrin, vitamin B6, purine, butanoate, and histidine, as well as aminoacyl tRNA biosynthesis (Figure 10C; Table 3). The integrated gene metabolite pathway network further showed the associations among DEGs, DEMs, and these pathways (Figure 10D).

### 3.7. α-Glucosidase Inhibition and Mechanism of Representative Flavonoids from XBL

**Figure 11:**
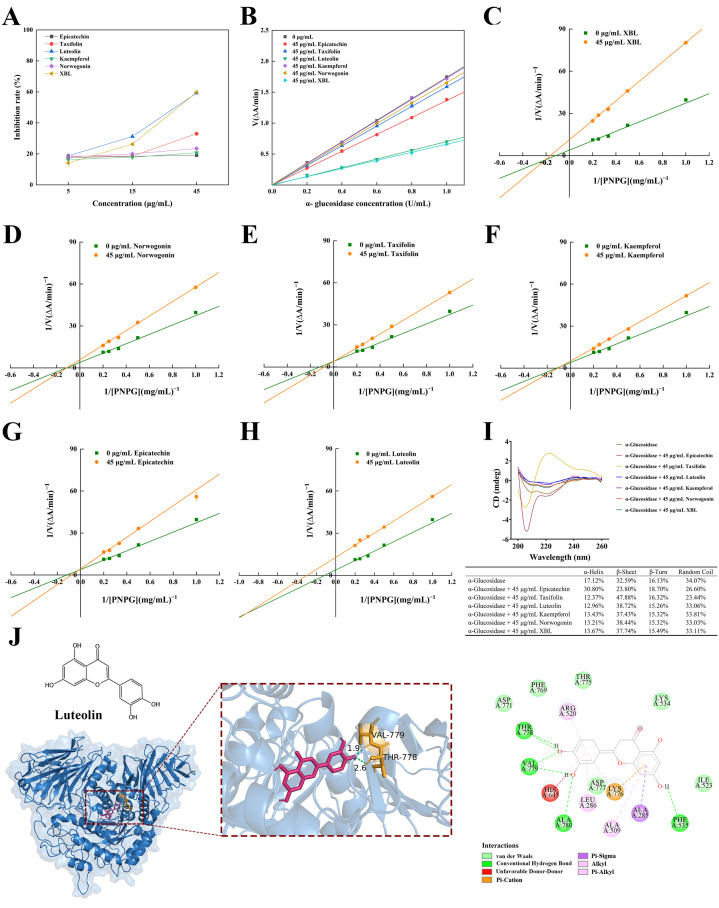
Inhibitory effects of XBL and its core components on α-glucosidase and their mechanisms. (**A**) Dose-dependent inhibition rates (%) of epicatechin, taxifolin, luteolin, kaempferol, norwogonin, and XBL (5, 15, 45 μg/mL); (**B**) Enzyme concentration versus reaction rate with and without 45 μg/mL inhibitor; (**C**–**H**) Lineweaver–Burk plots from kinetic analysis under 0 or 45 μg/mL inhibitor conditions; (**I**) Circular dichroism (CD) spectroscopy revealing changes in secondary structure composition, with small table showing percentage values for each group; (**J**) Chemical structure of luteolin and its binding mode with α-glucosidase.

As shown in Figure 11A, all tested samples inhibited α-glucosidase activity in a concentration dependent manner. Among the flavonoids, luteolin showed the strongest inhibition at 45 μg/mL. XBL showed comparable inhibitory activity, suggesting that representative flavonoids may contribute to its α-glucosidase inhibitory effect. To further assess their impacts on enzymatic activity, reaction rate was determined under different α-glucosidase concentrations in the absence or presence of inhibitors at 45 μg/mL. As shown in Figure 11B, reaction rate increased with α-glucosidase concentration in both the control system and inhibitor-containing systems. 

The fitted lines of XBL and representative flavonoids in Lineweaver–Burk plots were not parallel and mostly intersected on the left of the y-axis, suggesting mixed-type inhibition of α-glucosidase (Figure 11C–H). The higher slopes in the presence of inhibitors also indicated changes in the substrate-enzyme interaction.

The results of far-UV CD analysis are shown in Figure 11I. The free α-glucosidase displayed two characteristic peaks at 208 and 222 nm due to α-helix structures. However, the CD spectra and secondary structure composition of α glucosidase were changed after introduction of kaempferol, luteolin, epicatechin, taxifolin, norwogonin, and XBL. In detail, epicatechin was found to significantly increase the α-helix content. However, taxifolin, luteolin, kaempferol, norwogonin and XBL showed increases in β-sheet content relative to free α-glucosidase, indicating changes in the secondary structure of the enzyme after binding of these flavonoids.

The results of the molecular docking analysis are shown in Figure 11J. Luteolin showed the strongest α-glucosidase inhibition among the tested flavonoids and was selected for docking analysis. The docking results suggested that luteolin may bind to the α-glucosidase pocket through multiple interactions. Hydrogen bonding with THR 778 and van der Waals interactions with residues such as VAL 779 may contribute to the stability of the luteolin enzyme complex. These findings support the bioactivity of representative flavonoids from XBL and their potential contribution to the antidiabetic effect of the extract.

## 4. Discussion

Diabetic nephropathy (DN) is a major diabetes related microvascular complication and a leading cause of end stage renal disease. Previous studies have reported that conventional antidiabetic drugs may be limited by adverse effects and insufficient long term renal protection [17]. In this study, XBL improved glycemic status and attenuated renal injury in DN rats, suggesting its potential as a multi target dietary intervention for DN.

The protective role of XBL in DN was investigated by network pharmacology analysis, metabolomic profiling, and transcriptomic analysis. It is important to clarify that network pharmacology was used as a predictive approach to identify potential molecular targets rather than to provide direct mechanistic evidence. he results showed that XBL-related targets were enriched in pathways closely related to diabetic complications, like AGE–RAGE and HIF-1 signaling. These predicted pathways suggest possible molecular intersections linking hyperglycaemia-associated oxidative stress, inflammatory activation, and hypoxia-related responses in diabetic kidneys, thereby providing a plausible mechanistic framework for interpreting the multi-target actions of XBL. Consistent with these predictions, experimental validation in the STZ-induced DN model demonstrated that XBL administration alleviated characteristic pathological alterations, including mesangial expansion and glomerular sclerosis, and improved systemic metabolic parameters and renal injury indices [18]. Additionally, XBL treatment also attenuated diabetes-induced increases in serum lipids, BUN, Scr, Ucr, proteinuria, and kidney index.

These results indicate that XBL may preserve the glomerular filtration barrier function and maintaining podocyte structural and functional integrity [19]. Furthermore, the reduction in kidney index suggests that XBL may attenuate glomerulosclerosis by inhibiting the abnormal mesangial cell expansion and excessive extracellular matrix accumulation [20]. Collectively, these findings indicate the renoprotective effects of XBL induced by coordinated regulation of metabolic burden and renal tissue injury instead of the single target mechanism.

Glucose and lipid dysregulation are hallmarks of DN and contribute to renal oxidative stress, inflammatory activation, and progressive structural damage [21]. Hyperglycemia and lipid metabolic disorder and promote excessive ROS generation, which induce pro-inflammatory signaling pathways, including NF-κB, MAPK, and NLRP3 inflammasome pathways, thereby promoting the formation of glomerular and tubulointerstitial fibrosis, and impair renal mitochondrial function [22,23]. Renal metabolomics indicated that XBL restored the metabolic profile of diabetic kidney tissues, with differential metabolites mainly mapped to taurine and hypotaurine metabolism, galactose metabolism, and vitamin B6 metabolism. Taurine, a sulfur containing β amino acid, modulates renal inflammatory responses by suppressing NF-κB activation, reducing pro inflammatory cytokines such as TNF α, IL 6, and IL 1β, and promoting IL 10 release [24]. The coupling of inflammation and lipid metabolism pathways observed through multi-omics data aligns with findings that NF-κB/TNF-α signaling activation directly leads to renal lipid deposition, while PPARα restoration promotes fatty acid oxidation and counteracts lipotoxicity [25]. The simultaneous inhibition of TNF/NF-κB and upregulation of PPARα targets (e.g., Fabp1) by XBL indicate that it breaks this vicious “inflammation–lipotoxicity” cycle at the transcriptional level.

Additionally, its related metabolites such as betaine have also been reported to protect against DN by improving glucose and lipid metabolism, maintaining glomerular basement membrane integrity, preserving renal function, reducing oxidative stress, and suppressing inflammation [26]. In this study, the regulation of taurine related metabolites suggests that XBL enhanced antioxidant and anti-inflammatory responses in diabetic kidneys.

Galactose metabolism was also influenced after XBL treatment. D-galactose is prone to taking part in non-enzymatic glycation processes, thereby promoting the generation of advanced glycation end products (AGEs). Under diabetic conditions, AGE accumulation in renal tissues aggravates oxidative stress and inflammation and contributes to glomerular hypertrophy, nephrosclerosis, and albuminuria [27,28]. The interaction between galactose and the AGE-RAGE signaling pathway serves as a master regulator linking carbonyl stress to NF-κB-dependent inflammation; RAGE activation not only triggers NF-κB nuclear translocation and pro-inflammatory cytokine release [29], but also directly induces non-enzymatic glycation modifications of extracellular matrix proteins, thereby promoting mesangial expansion and glomerulosclerosis [30]. We observed that XBL normalized galactose metabolism while simultaneously downregulating RAGE-associated fibrotic genes, supporting its role in alleviating glycation-related renal injury via carbohydrate metabolism modulation. The regulation of galactose-related metabolites observed in this study supports that XBL may alleviate glycation-associated renal injury by modulating carbohydrate metabolism and reducing AGE-related metabolic stress. Disrupted galactose metabolism may promote the buildup of advanced glycation end products (AGEs), while vitamin B6 functions as an enzymatic cofactor and is essential for amino acid metabolic processes and antioxidant protection [31].

Additionally, XBL greatly influenced vitamin B6 metabolism, particularly pyridoxal phosphate, a coenzyme involved in amino acid metabolism with anti-glycation properties [32]. Hyperglycaemia-driven non-enzymatic protein glycation is a central pathogenic process in diabetic complications, and inhibition of glycation has been suggested as a therapeutic approach to mitigate diabetes-associated vascular and renal damage [33]. The restoration of pyridoxal phosphate content after XBL treatment indicates an enhanced endogenous anti-glycation activity, indicating that XBL may mitigate DN by regulating carbohydrate metabolism and glycation related pathways. Collectively, the metabolomic results suggest that XBL induces systems-level metabolic reprogramming in diabetic kidneys rather than correcting isolated metabolic abnormalities. Although MAPK was not the primary focus of our transcriptomic analysis, previous studies have confirmed that AGEs/RAGE/MAPK constitute a key signaling hub linking glycation stress to renal fibrosis [34]. Therefore, we hypothesize that the antioxidant metabolites restored by XBL (such as taurine and pyridoxal phosphate) may exert additional nephroprotective effects by inhibiting RAGE-downstream MAPK-mediated signaling.

Transcriptomic results indicated that XBL modulated several DN-related pathways including inflammation, fibrosis, and metabolic regulation, among which PPAR, TGF-β, and TNF were prominent [35]. Sustained activation of TGF-β signaling promotes extracellular matrix accumulation, mesangial expansion, and podocyte injury via downstream Smad-dependent mechanisms, ultimately inducing renal fibrosis and functional decline [36]. In this study, XBL suppressed the expression of TGF-β–associated genes, consistent with the attenuation of mesangial matrix accumulation and glomerulosclerosis in histopathological analysis. 

PPAR signaling links metabolic regulation with inflammatory control. PPARα activation promotes fatty acid oxidation and inhibits lipid deposition, while PPARγ regulates insulin responsiveness and contributes to anti-inflammatory actions in renal tissues [37]. Transcriptomic profiling indicated that XBL coordinately regulated PPAR-associated genes involved in ketogenesis (*Hmgcs2*), fatty acid transport (*Fabp1*), and lipid droplet dynamics (*Plin* family members). Coupled with the metabolomic evidence of improved lipid and carbohydrate metabolic profiles, these findings support that PPAR mediated metabolic rebalancing may contribute to downstream suppression of inflammatory and fibrotic responses. Additionally, the suppression of TNF-associated transcriptional responses indicated that XBL blocks inflammatory pathways that have been shown to be involved in podocyte dysfunction and DN progression [38], suggesting integrated metabolic and immune modulation by XBL.

Finally, integrated transcriptomic and metabolomic analysis provided further evidence of coordinated transcriptional and metabolic regulation. Notable associations were observed between 23 differential metabolites and 36 DEGs, suggesting close coordination between metabolic and transcriptional changes. Network topological analysis identified *Prc1*, *Igsf10*, SAICAR and D-galactose as major nodes in the gene metabolite network. 

Among the enriched pathways, galactose metabolism was the most prominent involved, indicating the involvement of carbohydrate metabolic dysregulation in DN progression. Disruption of galactose homeostasis may promote AGE accumulation and oxidative stress, exacerbating renal injury under diabetic conditions [39,40]. Additionally, enrichment of metabolic pathways related to tyrosine, taurine betaine, and histidine further suggests broader regulation of amino acid metabolism and redox balance related with DN [41]. Collectively, these integrated multi-omics findings support that XBL may restore renal homeostasis by coordinating metabolic remodeling and inflammation and fibrosis related signaling. 

To further validate the biological relevance of the predicted core constituents, representative flavonoids identified from XBL were evaluated by α-glucosidase activity analysis. The observed inhibitory activities, particularly for Luteolin, provide additional support that key flavonoids in XBL were functionally relevant bioactive components, thereby reinforcing the mechanistic interpretation derived from the in vivo pharmacodynamic and multi-omics analyses. This functional validation aligns with previous studies demonstrating that other plant-derived flavonoids, such as myricetin and quercetin, exert renoprotective effects in diabetic nephropathy models through the coordinated regulation of oxidative stress, inflammatory signaling, and metabolic reprogramming [42,43]. These findings place XBL and its flavonoid constituents within the broader context of flavonoid-based interventions for diabetic nephropathy.

Collectively, XBL greatly improved renal function and reduced histopathological injury, accompanied by regulation of metabolic pathways and suppression of inflammation-and fibrosis-related signaling. Validation of representative flavonoids also supported the biological relevance of main active constituents. Despite these findings, the absence of absolute quantification for major flavonoids warrants cautious interpretation of dose-dependent effects across studies. Future work will establish validated quantitative assays to enable standardized dosing and cross-laboratory reproducibility. Moreover, while network pharmacology prioritized plausible targets, definitive causal validation through genetic manipulation remains necessary to confirm these predicted mechanisms. Additionally, the current study did not perform qPCR or Western blot analyses; thus, the observed transcriptional and proteomic changes require further verification at the mRNA and protein expression levels. Finally, it should be noted that these effects were observed in a rat model, and species-specific metabolic differences may limit their direct translation to humans. However, it should be noted that these effects were observed in a rat model, and species-specific metabolic differences may limit their direct translation to humans.

## 5. Conclusions

In this study, the renoprotective properties of XBL against diabetic nephropathy were investigated using integrated network pharmacology analysis, in vivo evaluation, metabolomics, transcriptomics, and functional validation. XBL showed significant protective effects against diabetic nephropathy by alleviating renal dysfunction and histopathological injury, while coordinately modulating metabolic reprogramming and inflammation- and fibrosis-related signaling. Integrated network pharmacology, multi-omics analyses, and targeted functional validation further supported that these effects were associated with identifiable bioactive constituents and multi-target regulatory mechanisms. These findings provide systematic preclinical evidence supporting the biological plausibility of XBL as a candidate for further investigation in diabetic nephropathy. However, given the absence of human data, clinical translation requires comprehensive pharmacokinetic, safety, and efficacy evaluation in human populations before XBL can be considered for functional food or therapeutic application.

## Figures and Tables

**Table 1:** Topological parameters of XBL compounds in the compound–target network.

Compound	Degree Centrality	Betweenness Centrality	Closeness Centrality	Eigenvector Centrality
Epicatechin	84	1333.627753	0.001385042	0.090011595
Taxifolin	78	1066.609672	0.00136612	0.088499897
Norwogonin	68	1371.135873	0.001445087	0.206377659
Luteolin	65	1037.608094	0.001428571	0.201405793
Kaempferol	64	997.5370003	0.001428571	0.201928685

**Table 2:** Changes in differential metabolites under positive and negative modes.

Mode	No	Metabolite	Formula	Library ID	KEGG	*m*/*z*	Model vs. Control	XBL vs. Model	Related Pathway
ESI^−^	1	L-Glutamate	C_5_H_9_NO_4_	HMDB0000148	C00025	146.0462	↓ **	↑ ^#^	Arginine biosynthesis Porphyrin metabolism Alanine, aspartate and glutamate metabolism Histidine metabolism
	2	Sucrose	C_12_H_22_O_11_	HMDB0000258	C00089	387.1156	↑ ***	↓ ^###^	Galactose metabolism
	3	D-Galactose	C_6_H_12_O_6_	HMDB0000143	C00124	179.058	↑ ***	↓ ^###^	Galactose metabolism
	4	L-Histidine	C_6_H_9_N_3_O_2_	HMDB0000177	C00135	154.0628	↓ ***	↑	Histidine metabolism
	5	Taurine	C_2_H_7_NO_3_S	HMDB0000251	C00245	124.0075	↓ ***	↑ ^###^	Taurine and hypotaurine metabolism
	6	Pyridoxine 5′-phosphate	C_8_H_12_NO_6_P	HMDB0001319	C00627	248.0328	↓ ***	↑ ^#^	Vitamin B6 metabolism
	7	Porphobilinogen	C_10_H_14_N_2_O_4_	HMDB0000245	C00931	207.9694	↓ ***	↑ ^##^	Porphyrin metabolism
	8	FAICAR	C_13_H_19_N_4_O_12_P	HMDB0000797	C04823	435.0589	↓ ***	↑ ^###^	Purine metabolism
	9	5-L-Glutamyl-taurine	C_7_H_14_N_2_O_6_S	HMDB0004195	C05844	253.0502	↓ ***	↑	Taurine and hypotaurine metabolism
ESI^+^	10	Pyridoxal 5′-phosphate	C_8_H_10_NO_6_P	HMDB0001491	C00018	248.1494	↑ **	↓ ^##^	Vitamin B6 metabolism
	11	Tyrosine	C_9_H_11_NO_3_	HMDB0000158	C00082	182.0811	↓ ***	↑ ^##^	Tyrosine metabolism
	12	GMP	C_10_H_14_N_5_O_8_P	HMDB0001397	C00144	320.0742	↑ ***	↓ ^###^	Purine metabolism
	13	Carbamoyl phosphate	CH_4_NO_5_P	HMDB0001096	C00169	159.0166	↓ **	↓ ^##^	Arginine biosynthesis Alanine, aspartate and glutamate metabolism
	14	Adenosine	C_10_H_13_N_5_O_4_	HMDB0000050	C00212	268.1035	↑ ***	↓ ^###^	Purine metabolism
	15	Deoxyguanosine	C_10_H_13_N_5_O_4_	HMDB0000085	C00330	224.1134	↑ *	↓ ^##^	Purine metabolism
	16	4-Aminobutanoate (GABA)	C_4_H_9_NO_2_	HMDB0000112	C00334	104.0707	↓ *	↓ ^#^	Alanine, aspartate and glutamate metabolism
	17	Guanosine	C_10_H_13_N_5_O_5_	HMDB0000133	C00387	284.0986	↓ *	↑	Purine metabolism
	18	D-Sorbitol	C_6_H_14_O_6_	HMDB0000247	C00794	166.0582	↓ ***	↑ ^##^	Galactose metabolism
	19	Hydroxymethylbilane	C_40_H_46_N_4_O_17_	HMDB0001137	C01024	893.3713	↓ ***	↑ ^###^	Porphyrin metabolism
	20	4-Fumarylacetoacetate	C_8_H_8_O_6_	HMDB0001268	C01061	218.1029	↑ ***	↓ ^###^	Tyrosine metabolism
	21	Protoporphyrinogen IX	C_34_H_40_N_4_O_4_	HMDB0001097	C01079	552.2985	↓ **	↑ ^##^	Porphyrin metabolism
	22	Dopamine	C_8_H_11_NO_2_	HMDB0000073	C03758	136.0756	↓ ***	↑ ^#^	Tyrosine metabolism
	23	SAICAR	C_10_H_15_N_4_O_9_P	HMDB0001439	C04734	405.1524	↑ *	↓ ^###^	Purine metabolism
	24	3,4-Dihydroxymandelate	C_8_H_8_O_5_	HMDB0001866	C05580	141.0547	↑ ***	↓ ^###^	Tyrosine metabolism

↑ indicates increase; ↓ indicates decrease. * *p* < 0.05; ** *p* < 0.01; *** *p* < 0.001 vs. Control group ^#^ *p* < 0.05; ^##^ *p* < 0.01; ^###^ *p* < 0.001 vs. Model group.

**Table 3:** Top 15 metabolic pathways enriched in DN rats.

Pathway	*p*-Value	Impact Factor
Galactose metabolism	0.0020053	0.5
Tyrosine metabolism	0.014178	0.25287
Taurine and hypotaurine metabolism	0.014191	0.375
Porphyrin metabolism	0.021031	0.092593
Vitamin B6 metabolism	0.021332	0.35
Purine metabolism	0.036599	0.15116
Butanoate metabolism	0.039089	0.14286
Aminoacyl-tRNA biosynthesis	0.045287	0.041096
Histidine metabolism	0.046807	0.22581
Glycerolipid metabolism	0.05224	0.24242
Nitrogen metabolism	0.10443	0.22222
Phenylalanine, tyrosine and tryptophan biosynthesis	0.11428	0.6
Phenylalanine metabolism	0.20717	0.05
Arginine biosynthesis	0.26653	0.18519
Ubiquinone and other terpenoid-quinone biosynthesis	0.31399	0.030303

## Data Availability

The original contributions presented in the study are included in the article, further inquiries can be directed to the corresponding author.

## Supplementary Materials

The following supporting information can be downloaded at: https://www.mdpi.com/article/10.3390/foods15142576/s1, Figure S1: Total ion chromatograms (TIC) of XBL extract in positive (A) and negative (B) ion modes obtained by UPLC-MS; Figure S2: KEGG pathway mapping of DEGs affected by XBL treatment. DEGs enriched in the PPAR signaling pathway (A), TGF-β signaling pathway (B), and TNF signaling pathway (C) are visualized using the KEGG pathway database. The color scale represents gene expression changes (log_2_ fold change), with red indicating up-regulation and blue indicating down-regulation; Table S1: Summary of the 293 chemical constituents tentatively identified in XBL; Table S2: List of 18 candidate bioactive compounds meeting the criteria of OB ≥ 20% and DL ≥ 0.18; Table S3: Pearson correlation coefficients between DEGs and DEMs; Table S4: Raw metabolomics profiling data of kidney tissues; Method S1: Determination of α-glucosidase inhibitory activity of XBL and representative core flavonoids; Method S2: Enzyme kinetic analysis of representative flavonoids against α-glucosidase; Method S3: Circular dichroism spectroscopy of α-glucosidase in the presence of representative flavonoids and XBL; Method S4: Molecular docking analysis of luteolin with α-glucosidase.

## Abbreviations

The following abbreviations are used in this manuscript:

DN	diabetic nephropathy
XBL	*Xanthoceras sorbifolium* Bunge leaves
STZ	streptozotocin
SD	Sprague-Dawley
FBG	fasting blood glucose
ESRD	end-stage renal disease
RAAS	renin–angiotensin–aldosterone system
GLP-1RAs	glucagon-like peptide-1 receptor agonists
SGLT-2	sodium-glucose cotransporter-2 inhibitors
TC	total cholesterol
TG	triglycerides
PRO	urine protein
Cr	creatinine
BUN	blood urea nitrogen
PPI	protein–protein interaction
BC	betweenness centrality
CC	closeness centrality
DC	degree centrality
EC	eigenvector centrality
LC-MS	liquid chromatography–mass spectrometry
H&E	Hematoxylin–eosin
PCA	principal component analysis
PLS-DA	partial least squares discriminant analysis
OPLS-DA	orthogonal partial least squares discrimination analysis
HMDB	Human Metabolome Database
KEGG	Kyoto Encyclopedia of Genes and Genomes
DEGs	differentially expressed genes
AGEs	advanced glycation end products
