# Supplementary material for: Xanthoceras sorbifolium Bunge Leaf Extract Ameliorates Diabetic Nephropathy Through Coordinated Metabolic Reprogramming and Inflammatory Signaling
Source: Foods. 2026 Jul 22;15(14):2576. doi: 10.3390/foods15142576 (PMC13409500; doi:10.3390/foods15142576)
Supplement: Supplementary file 1 [file foods-15-02576-s001.zip › Supplementary Material.pdf]

Supplemental information:

Xanthoceras sorbifolium Bunge leaf extract ameliorates diabetic nephropathy through coordinated metabolic reprogramming and inflammatory signaling

Mengting Han[1], Xianyu Zhang[1], Yiqing Jia[1], Yifei Zhang[1], Zijin Qin[2], Shuyu Zhou[1], Zhe Xu[1,*], Hui Zhou[1,*]

[1] Key Laboratory of Biotechnology and Bioresources Utilization, Ministry of Education, Dalian Minzu University, Dalian 116600, China

[2] Department of Food Science and Technology, University of Georgia, Athens, GA 30602, USA

*Corresponding author:

Zhe Xu, E-mail: xuzhe@dlnu.edu.cn

Hui Zhou, E-mail: zhouhui@dlnu.edu.cn

Method S1. Determination of α-glucosidase inhibitory activity of XBL and representative core flavonoids

The α-glucosidase inhibitory activities of XBL and representative core flavonoids were determined using a colorimetric assay with p-nitrophenyl-α-D-glucopyranoside (p-NPG) as the substrate. Briefly, α-glucosidase solution (1 U/mL) was incubated with kaempferol, luteolin, epicatechin, taxifolin, norwogonin, or XBL at final concentrations of 5, 15, and 45 μg/mL in 0.2 M PBS (pH 7.0) at 37 °C for 10 min. Subsequently, 5 mM p-NPG was added to initiate the reaction, and the mixture was further incubated at 37 °C for 30 min. The reaction was terminated by adding 180 μL of 0.2 M Na_2CO_3, and the absorbance was recorded at 405 nm using a Synergy H1 microplate reader. Acarbose was used as the positive control.

The inhibitory rate (I%) was calculated according to Eq. (1):

$$I\% = [1-(B-b)/(A-a)] \times 100\% \tag{1}$$

where A is the absorbance of the control group, a is the absorbance of the control blank group, B is the absorbance of the sample group, and b is the absorbance of the sample blank group.

Method S2: Enzyme kinetic analysis of representative flavonoids against α-glucosidase

To further characterize the inhibitory behavior of representative flavonoids against α-glucosidase, enzyme kinetic analysis was performed using p-NPG as the substrate. For reaction rate analysis under different enzyme concentrations, p-NPG solution (5 mM) was incubated with kaempferol, luteolin, epicatechin, taxifolin, norwogonin, or XBL at 45 μg/mL at 37 °C for 15 min. α-Glucosidase solutions at different concentrations (1.0, 0.8, 0.6, 0.4, and 0.2 U/mL) were then added to initiate the reaction, and the absorbance was measured at 405 nm. Enzyme activity was estimated from the change in absorbance, and reaction rate curves were plotted against enzyme concentration.

For Lineweaver–Burk analysis, p-NPG was prepared at concentrations ranging

from 1 to 5 mM, while the inhibitor concentration was fixed at 45 μg/mL. The initial reaction velocities were determined at 37 °C, and double-reciprocal plots were constructed by plotting 1/v against 1/[S], where v is the initial reaction velocity and [S] is the substrate concentration. The inhibition type was inferred from the changes in slope and intercept of the fitted lines in the presence or absence of inhibitors.

Method S3. Circular dichroism spectroscopy of α-glucosidase in the presence of representative flavonoids and XBL

Circular dichroism (CD) spectroscopy was performed to assess the effects of representative flavonoids and XBL on the secondary structure of α-glucosidase. α-glucosidase solution (1 U/mL) was incubated with kaempferol, luteolin, epicatechin, taxifolin, norwogonin, or XBL at a final concentration of 45 μg/mL. The CD spectra were recorded in the range of 200–260 nm with a bandwidth of 1 nm and a scan rate of 100 nm/min. PBS buffer (0.2 M, pH 7.0) was used as the background control, and the spectra were analyzed using CDNN 2.1 software.

Method S4. Molecular docking analysis of luteolin with α-glucosidase

Molecular docking was performed to predict the binding interaction between luteolin and α-glucosidase using AutoDockTools software (V1.5.7). The crystal structure of α-glucosidase was obtained from the Protein Data Bank (PDB ID: 2QMJ), and the three-dimensional structure of luteolin was retrieved from the PubChem database (CID: 5280445). The docking poses were generated using AutoDock Vina, and the interaction patterns were analyzed and visualized using PyMOL and Discovery Studio.

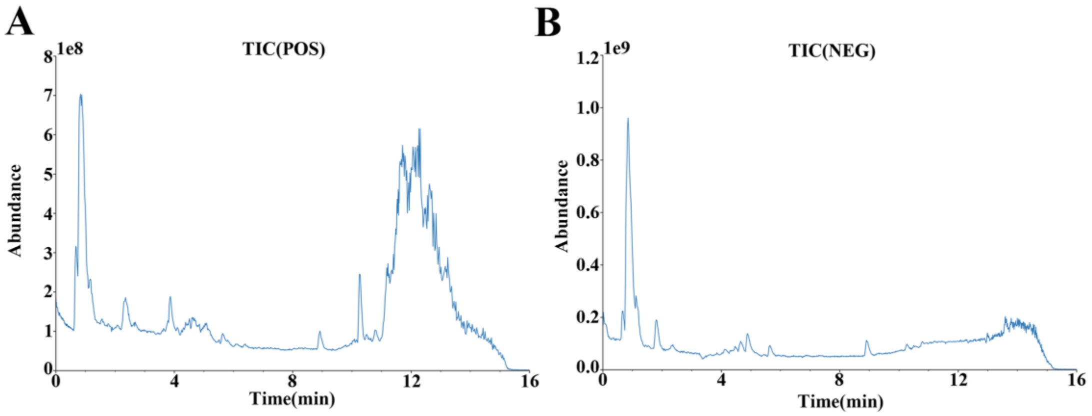

Figure S1. Total ion chromatograms (TIC) of XBL extract in positive (A) and negative (B) ion modes obtained by UPLC-MS.

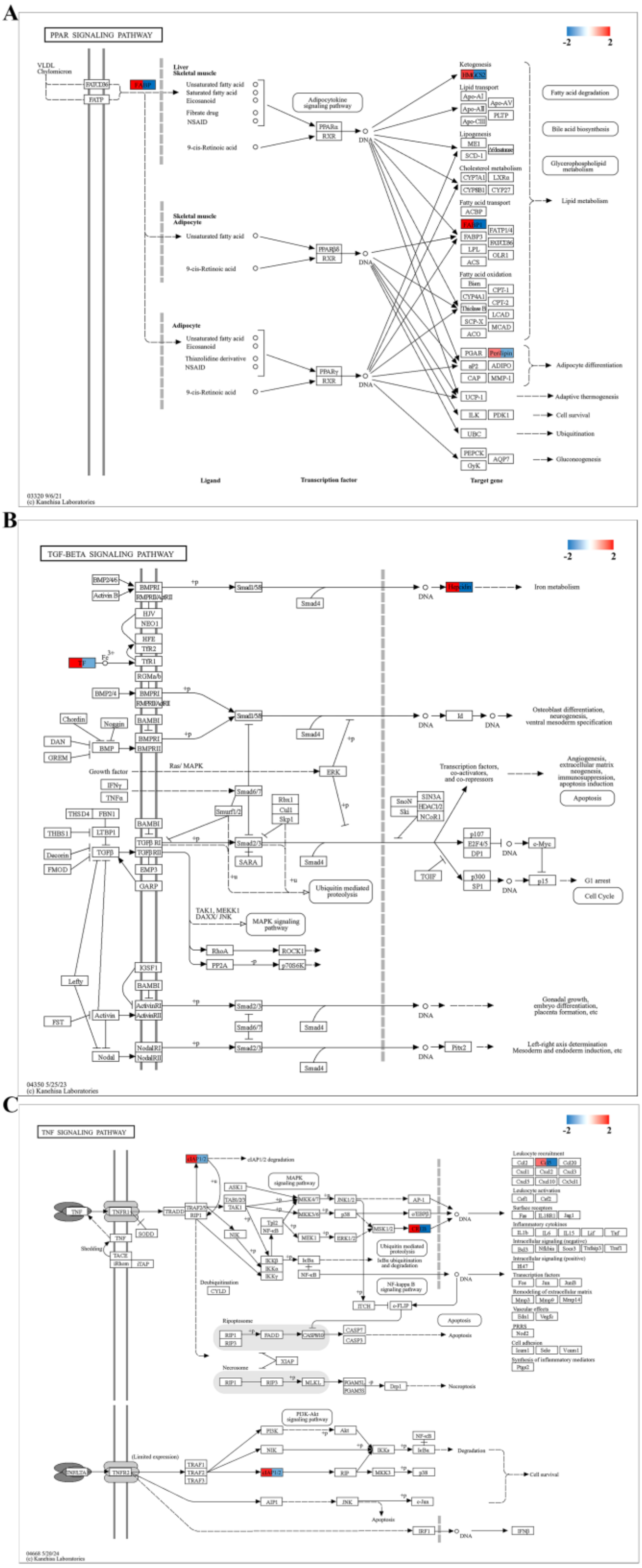

Figure S2. KEGG pathway mapping of DEGs affected by XBL treatment. DEGs enriched in the PPAR signaling pathway (A), TGF-β signaling pathway (B), and TNF signaling pathway (C) are visualized using the KEGG pathway database. The color scale represents gene expression changes (log_2 fold change), with red indicating up-regulation and blue indicating down-regulatio

Table S1. Summary of the 293 chemical constituents tentatively identified in XBL.

No.	Compounds	Formula	RT/min
M0001	5-Hydroxymethyl-2-furancarboxaldehyde	$C_6H_6O_3$	2.664
M0002	3-Hydroxy-2-Methylpyridine	C_6H_7NO	1.121
M0003	Betaine	$C_5H_{11}NO_2$	0.849
M0004	Isonicotinamide	$C_6H_6N_2O$	1.149
M0005	Maltol	$C_6H_6O_3$	3.722
M0006	Levoglucosan	$C_6H_{10}O_5$	0.831
M0007	Pipecolic acid	$C_6H_{11}NO_2$	0.958
M0008	Trigonelline	$C_7H_7NO_2$	0.865
M0009	Stachydrine	$C_7H_{13}NO_2$	0.89
M0010	(E)-m-Coumaric acid	$C_9H_8O_3$	4.943
M0011	Gallic acid	$C_7H_6O_5$	1.821
M0012	L-Glucose	$C_6H_{12}O_6$	0.819
M0013	(2R,3R,4S,5R)-2-Amino-3,4,5,6-tetrahydroxyhexanal	$C_6H_{13}NO_5$	0.846
M0014	3-(3,4-Dimethoxyphenyl)-2-propenoic acid	$C_{11}H_{12}O_4$	6.058
M0015	Fructose	$C_6H_{12}O_6$	0.827
M0016	purpurogallin	$C_{11}H_8O_5$	5.792
M0017	D-ononitol	$C_7H_{14}O_6$	0.846
M0018	Spongouridine	$C_9H_{12}N_2O_6$	1.216
M0019	Cytarabine	$C_9H_{13}N_3O_5$	0.886
M0020	Armillarisin A	$C_{12}H_{10}O_5$	5.076
M0021	3,4,8,9,10-Pentahydroxy Urolithin	$C_{13}H_8O_7$	4.677
M0022	Vidarabine	$C_{10}H_{13}N_5O_4$	1.267
M0023	Petroselinic acid	$C_{18}H_{34}O_2$	13.793

M0024	Kaempferol	$C_{15}H_{10}O_6$	7.028
M0025	Brevifolincarboxylic acid	$C_{13}H_8O_8$	4.292
M0026	Ellagic acid	$C_{14}H_6O_8$	4.936
M0027	Morin	$C_{15}H_{10}O_7$	6.203
M0028	(2R,3R,4S,5S,6R)-2-[(2E)-4-hydroxy-3,7-dimethylocta-2,6-dienoxy]-6-(hydroxymethyl)oxane-3,4,5-triol	$C_{16}H_{28}O_7$	4.84
M0029	L-Ascorbic acid 2-glucoside	$C_{12}H_{18}O_{11}$	0.976
M0030	Turanose	$C_{12}H_{22}O_{11}$	0.849
M0031	Cis-Ferulic acid 4-O-beta-D-glucopyranoside	$C_{16}H_{20}O_9$	4.355
M0032	Ferulic acid 4-glucoside	$C_{16}H_{20}O_9$	4.057
M0033	8-O-Acetylharpagide	$C_{17}H_{26}O_{11}$	4.527
M0034	Hastatoside	$C_{17}H_{24}O_{11}$	4.29
M0035	Kaempferol 7-rhamnoside	$C_{21}H_{20}O_{10}$	5.992
M0036	Roseoside	$C_{19}H_{30}O_8$	4.506
M0037	Asperuloside acid	$C_{18}H_{24}O_{12}$	3.763
M0038	Quercetin 7-rhamnoside	$C_{21}H_{20}O_{11}$	5.448
M0039	Gaultherin	$C_{19}H_{26}O_{12}$	4.503
M0040	Astragalin	$C_{21}H_{20}O_{11}$	5.095
M0041	Isorhamnetin 7-rhamnoside	$C_{22}H_{22}O_{11}$	6.094
M0042	Isoquercitrin	$C_{21}H_{20}O_{12}$	4.895
M0043	Isorhamnetin 3-glucoside	$C_{22}H_{22}O_{12}$	5.146
M0044	Quercetin 3-(6"-malonylglucoside)	$C_{24}H_{22}O_{15}$	4.887
M0045	Manninotriose	$C_{18}H_{32}O_{16}$	0.868
M0046	Pinoresinol 4-O-beta-D-glucopyranoside	$C_{26}H_{32}O_{11}$	5.082
M0047	[(2R,3S,6S)-6-[5,7-Dihydroxy-2-(4-hydroxyphenyl)-4-oxochromen-3-yl]oxy-3,4,5-trihydroxyoxan-2-yl]methyl 3-(4-hydroxyphenyl)prop-2-enoate	$C_{30}H_{26}O_{13}$	5.928
M0048	Luteolin 7-neohesperidoside	$C_{27}H_{30}O_{15}$	4.636

M0049	Quercetin 3-neohesperidoside	$C_{27}H_{30}O_{16}$	4.741
M0050	Kaempferol 3,7-diglucoside	$C_{27}H_{30}O_{16}$	4.248
M0051	Narcissin	$C_{28}H_{32}O_{16}$	4.962
M0052	Quercilicoside A	$C_{36}H_{58}O_{11}$	5.544
M0053	Kaempferol 3-sophoroside-7-rhamnoside	$C_{33}H_{40}O_{20}$	4.248
M0054	Quercetin 3-(2Gal-rhamnosyl-robinobioside)	$C_{33}H_{40}O_{20}$	4.485
M0055	Isorhamnetin 3-sophoroside-7-rhamnoside	$C_{34}H_{42}O_{21}$	4.292
M0056	Isorhamnetin 3-glucoside-7-rhamnoside	$C_{28}H_{32}O_{16}$	4.672
M0057	Maltohexaose	$C_{36}H_{62}O_{31}$	0.892
M0058	beta-Cyclodextrin	$C_{42}H_{70}O_{35}$	0.917
M0059	gamma-Cyclodextrin	$C_{48}H_{80}O_{40}$	1.11
M0060	Isomaltotetraose	$C_{24}H_{42}O_{21}$	0.812
M0061	Maltopentaose	$C_{30}H_{52}O_{26}$	0.831
M0062	Glycolaldehyde dimer	$C_4H_8O_4$	0.819
M0063	D-Leucic acid	$C_6H_{12}O_3$	4.758
M0064	D-threonic acid	$C_4H_8O_5$	0.856
M0065	Glycylglycine	$C_4H_8N_2O_3$	0.821
M0066	Xanthopterin	$C_6H_5N_5O_2$	0.987
M0067	Fagomine	$C_6H_{13}NO_3$	0.846
M0068	Mesalazine	$C_7H_7NO_3$	0.904
M0069	Methyl 5-hydroxypyridine-2-carboxylate	$C_7H_7NO_3$	4.332
M0070	3-Methyl-2-oxovaleric acid	$C_6H_{10}O_3$	4.19
M0071	Fosfomycin	$C_3H_7O_4P$	0.897
M0072	Sesamol	$C_7H_6O_3$	4.292
M0073	Arecaidine	$C_7H_{11}NO_2$	0.685
M0074	Indole-3-carbinol	C_9H_9NO	4.949

M0075	Galacturonic acid	$C_6H_{10}O_7$	0.955
M0076	L-Arabinitol	$C_5H_{12}O_5$	0.827
M0077	4-Hydroxycoumarin	$C_9H_6O_3$	5.482
M0078	Castanospermine	$C_8H_{15}NO_4$	0.86
M0079	Carglumic acid	$C_6H_{10}N_2O_5$	0.831
M0080	Genipin	$C_{11}H_{14}O_5$	4.76
M0081	Aminovaleric acid betaine	$C_8H_{17}NO_2$	0.865
M0082	beta-D-N-Acetylmannosamine	$C_8H_{15}NO_6$	0.87
M0083	Allitol	$C_6H_{14}O_6$	0.812
M0084	Sinapic acid	$C_{11}H_{12}O_5$	5.246
M0085	Gulonolactone	$C_6H_{10}O_6$	0.918
M0086	Canavanine	$C_5H_{12}N_4O_3$	1.008
M0087	Nicotinic acid riboside	$C_{11}H_{13}NO_6$	1.152
M0088	Mannoheptulose	$C_7H_{14}O_7$	0.917
M0089	Vomifoliol	$C_{13}H_{20}O_3$	5.244
M0090	Bombykol	$C_{16}H_{30}O$	12.655
M0091	(1R,4E,9E,11S)-4,12,12-trimethyl-8-oxobicyclo[9.1.0]dodeca-4,9-dien-2-yl acetate	$C_{17}H_{24}O_3$	10.516
M0092	Kifunensine	$C_8H_{12}N_2O_6$	5.46
M0093	Vicine	$C_{10}H_{16}N_4O_7$	0.897
M0094	5-Hydroxymethyldeoxycytidine monophosphate	$C_{10}H_{15}N_3O_5$	0.794
M0095	Pinobanksin 3-acetate	$C_{17}H_{14}O_6$	8.986
M0096	Xylobiose	$C_{10}H_{18}O_9$	0.963
M0097	Kinsenoside	$C_{10}H_{16}O_8$	0.955
M0098	tubercidin	$C_{11}H_{14}N_4O_4$	0.868
M0099	Norwogonin	$C_{15}H_{10}O_5$	7.215
M0100	1-O-alpha-D-Glucopyranosyl-D-mannitol	$C_{12}H_{24}O_{11}$	0.937

M0101	Kaurenoic acid	$C_{20}H_{30}O_2$	12.992
M0102	Chebulic acid	$C_{14}H_{12}O_{11}$	1.183
M0103	Lactobionic acid	$C_{12}H_{22}O_{12}$	0.856
M0104	8-epideoxyloganic acid	$C_{16}H_{24}O_9$	4.524
M0105	[6]-Gingerdiol 3,5-diacetate	$C_{21}H_{32}O_6$	10.996
M0106	Secoxyloganin	$C_{17}H_{24}O_{11}$	4.909
M0107	Methylsyringin	$C_{18}H_{26}O_9$	5.009
M0108	Obacunone	$C_{26}H_{30}O_7$	9.966
M0109	Xylotriose	$C_{15}H_{26}O_{13}$	0.907
M0110	Alisol B	$C_{30}H_{48}O_4$	12.288
M0111	Withanolide A	$C_{28}H_{38}O_6$	8.939
M0112	Maslinic acid	$C_{30}H_{48}O_4$	11.258
M0113	Epigallocatechin 3-O-(3-O-methylgallate)	$C_{23}H_{20}O_{11}$	4.7
M0114	Arjunolic acid	$C_{30}H_{48}O_5$	9.427
M0115	Vicenin 3	$C_{26}H_{28}O_{14}$	4.677
M0116	Sibiricose A5	$C_{22}H_{30}O_{14}$	4.506
M0117	25-methoxyalisol A	$C_{31}H_{52}O_5$	11.89
M0118	Secoisolariciresinol monoglucoside	$C_{26}H_{36}O_{11}$	4.962
M0119	Luteolin 7-apiosyl-(1->2)-glucoside	$C_{26}H_{28}O_{15}$	4.55
M0120	23(R)-16beta-hydroperoxyalisol B 23-acetate	$C_{32}H_{50}O_7$	10.516
M0121	Xylotetraose	$C_{20}H_{34}O_{17}$	0.936
M0122	1,4-beta-Xylopentaose	$C_{25}H_{42}O_{21}$	0.918
M0123	Kaempferol 3-sophoroside-7-glucoside	$C_{33}H_{40}O_{21}$	4.127
M0124	(+)-Syringaresinol O-beta-D-glucoside	$C_{28}H_{36}O_{13}$	5.096
M0125	2-O-beta-L-galactopyranosylorientin	$C_{27}H_{30}O_{16}$	4.472
M0126	Fructo-oligosaccharide DP7/GF6	$C_{42}H_{72}O_{36}$	0.856

M0127	Fructo-oligosaccharide DP8/GF7	$C_{48}H_{82}O_{41}$	0.856
M0128	Fructo-oligosaccharide DP9/GF8	$C_{54}H_{92}O_{46}$	0.803
M0129	5-methylol-3H-furan-2-one	$C_5H_6O_3$	0.831
M0130	Pyrogallol	$C_6H_6O_3$	2.174
M0131	1,2,4-Trihydroxybenzene	$C_6H_6O_3$	1.821
M0132	Malic acid	$C_4H_6O_5$	0.976
M0133	6-(hydroxymethyl)pyridin-3-ol	$C_6H_7NO_2$	0.91
M0134	4-hydroxybenzoate	$C_7H_5O_3$	5.806
M0135	Methyl 3-furoate	$C_6H_6O_3$	0.879
M0136	DL-Pyroglutamic acid	$C_5H_7NO_3$	1.21
M0137	Glycerophosphoric acid	$C_3H_9O_6P$	0.856
M0138	Aspartic acid	$C_4H_7NO_4$	0.794
M0139	Quinone	$C_6H_4O_2$	3.46
M0140	(1R,2R,3S)-3-methylcyclopentane-1,2-diol	$C_6H_{12}O_2$	4.292
M0141	threono-1,4-lactone	$C_4H_6O_4$	1.016
M0142	2,6,10-trimethyl-dodecane	$C_6H_8O_4$	2.036
M0143	2-(2,3,4-trihydroxyphenyl)acetonitrile	$C_8H_7NO_3$	3.468
M0144	2,3,4,5-tetrahydroxypentanoic acid	$C_5H_{10}O_6$	0.836
M0145	DL-3-Phenyllactic acid	$C_9H_{10}O_3$	5.082
M0146	Norhygrine	$C_7H_{13}NO$	4.8
M0147	dehydroascorbicacid	$C_6H_6O_6$	1.143
M0148	p-hydroxy-coumaric acid	$C_9H_{10}O_4$	3.927
M0149	4,4-dimethyl-1,7-heptanedioicacid	$C_9H_{16}O_4$	5.466
M0150	Cinchoninic acid, 2-hydroxy-	$C_{10}H_7NO_3$	4.078
M0151	N-Acetyl-DL-glutamic acid	$C_7H_{11}NO_5$	1.268
M0152	3,5,7-Trihydroxy chromone	$C_9H_6O_5$	4.399

M0153	(2S,3S,4S,5R)-2,3,4,5,6-pentahydroxyhexanoic acid	$C_6H_{12}O_7$	0.836
M0154	Benzeneacetic acid, 4-hydroxy-, ethyl ester	$C_{10}H_{12}O_3$	6.118
M0155	Metacetamol	$C_8H_9NO_2$	4.227
M0156	D-Tyrosine	$C_9H_{11}NO_3$	1.152
M0157	2,4,6-Cycloheptatrien-1-one, 2-hydroxy-3-(1-methylethyl)-	$C_{10}H_{12}O_2$	1.373
M0158	2-Hydroxy-2,6,6-trimethylcyclohexanone	$C_9H_{16}O_2$	6.122
M0159	ZINC00402871	$C_8H_{10}O_4$	1.149
M0160	nukagenin	$C_{10}H_7NO_4$	3.57
M0161	corydaldine(tautomericstructure 1)	$C_{11}H_{13}NO_3$	5.062
M0162	1-[2-(furan-2-yl)-2-oxoethyl] pyrroli-din-2-one	$C_{10}H_{11}NO_3$	4.61
M0163	Gularic acid	$C_6H_{10}O_8$	0.856
M0164	Coumaric acid (isomer of 131)	$C_9H_8O_3$	4.861
M0165	isololiolide	$C_{11}H_{16}O_3$	5.464
M0166	DL-Dopa	$C_9H_{11}NO_4$	1.17
M0167	N-(3,4,5-trimethoxyphenyl)formamide	$C_{10}H_{13}NO_4$	4.526
M0168	myo-Inositol	$C_6H_{12}O_6$	0.847
M0169	olibanumol,b	$C_{10}H_{18}O_2$	6.899
M0170	Droserone	$C_{11}H_8O_4$	5.731
M0171	1-Deoxyeucommiol	$C_9H_{16}O_3$	5.061
M0172	Octopinic acid	$C_8H_{16}N_2O_4$	0.87
M0173	7-hydroxy-6-(2-hydroxyethyl)coumarin	$C_{11}H_{10}O_4$	5.57
M0174	ethyl p-methoxy-cis-cinnamate	$C_{12}H_{14}O_3$	5
M0175	arteamisinine i	$C_{13}H_{18}O_2$	4.504
M0176	3,4-Dihydroxy-5-[(S)-1,2-dihydroxyethyl]furan-2(5H)-one	$C_6H_8O_6$	0.897
M0177	Erythrocentaurin	$C_{10}H_8O_3$	5.493
M0178	Pemoline	$C_9H_8N_2O_2$	3.588

M0179	3-Buten-2-one,4-(4-hydroxy-2,6,6-trimethyl-1-cyclohexen-1-yl)-, (3E)-	$C_{13}H_{20}O_2$	4.142
M0180	Genipinic acid	$C_{11}H_{14}O_6$	4.781
M0181	Methyl acetylsalicylate	$C_{10}H_{10}O_4$	1.17
M0182	Methyl-alpha-D-fructofuranoside	$C_7H_{14}O_6$	0.865
M0183	dimethyl camphorate	$C_{12}H_{20}O_4$	7.315
M0184	cis-1-(2-furyl)-4-(2-thienyl)-1-buten-3-yne	$C_{12}H_8OS$	5.668
M0185	5,6-dimethoxy-n-methylphthalimide	$C_{11}H_{11}NO_4$	5.731
M0186	regaloside D_qt	$C_{12}H_{14}O_5$	4.798
M0187	6'-O-cinnamoylharpagirle_qt 2	$C_{10}H_{16}O_4$	4.451
M0188	2-[(1R)-4-methyl-1-cyclohex-3-enyl]prop-2-enyl acetate	$C_{12}H_{18}O_2$	9.82
M0189	Wilfordic acid	$C_{11}H_{13}NO_4$	5.246
M0190	Cyclo(Pro-Val)	$C_{10}H_{16}N_2O_2$	4.592
M0191	Myristamide	$C_{14}H_{29}NO$	11.567
M0192	2,3,4-Trihydroxy-benzenepropanoic acid	$C_9H_{10}O_5$	2.917
M0193	methyl 2-hydroxy-3,4-dimethoxybenzoate	$C_{10}H_{12}O_5$	1.315
M0194	Ganoderpurine	$C_{11}H_{15}N_5O$	0.87
M0195	annuionone e	$C_{13}H_{22}O_3$	7.155
M0196	Tectoquinone	$C_{15}H_{10}O_2$	3.931
M0197	(e)-2-hexenyl-beta-d-glucopyranoside	$C_{12}H_{22}O_6$	5.174
M0198	1,2,3-Propanetricarboxylic acid, 2-hydroxy-, trimethyl ester	$C_9H_{14}O_7$	0.849
M0199	cheliensisamine	$C_{12}H_9NO_4$	3.889
M0200	carbalexin c	$C_{14}H_{13}NO_2$	6.18
M0201	(2R,3R,4S)-2-(6-aminopurin-9-yl)-4-(hydroxymethyl)oxolan-3-ol	$C_{10}H_{13}N_5O_3$	1.152
M0202	Tetradecyl acetate	$C_{16}H_{32}O_2$	8.123
M0203	Deoxyeritadenine	$C_9H_{11}N_5O_3$	1.397
M0204	2,3-Butanediol glucoside	$C_{10}H_{20}O_7$	1.632

M0205	lilioside C	$C_9H_{18}O_8$	0.849
M0206	2-Ethyl-2,4,5-trimethyl-1,3-dioxolane	$C_8H_{16}O_2$	7.703
M0207	9-Octadecenamide	$C_{18}H_{35}NO$	12.906
M0208	Methyl 2-[(1R,4R,4aS,8aR)-4,7-dimethyl-1,2,3,4,4a,5,6,8a-octahydronaphthalen-1-yl]prop-2-enoate	$C_{16}H_{24}O_2$	9.313
M0209	Sterol	$C_{17}H_{28}O$	11.166
M0210	9-hydroxy-10,12-Octadecadienoic acid	$C_{18}H_{32}O_3$	10.918
M0211	(12R)-12-Hydroxyoctadec-9-enoic acid	$C_{18}H_{34}O_3$	11.149
M0212	6-O-malonyl-beta-methyl-D-glucopyranoside	$C_{10}H_{16}O_9$	0.924
M0213	Bungeiside B	$C_{14}H_{18}O_8$	4.205
M0214	xanthienopyran	$C_{17}H_{16}O_4S$	2.219
M0215	Benzoic acid + 2O, O-Hex	$C_{13}H_{16}O_9$	2.846
M0216	(2S,3R,4S,5S,6R)-2-(benzyloxy)-6-methylol-tetrahydropyran-3,4,5-triol	$C_{13}H_{18}O_6$	4.42
M0217	9,10-dihydroxystearic acid	$C_{18}H_{36}O_4$	10.501
M0218	dimethyl 2-hydroxybutanedioate	$C_6H_{10}O_5$	1.545
M0219	Coumaroyl Hexoside (isomer of 691, 692)	$C_{15}H_{18}O_8$	4.227
M0220	6-Galloylglucose	$C_{13}H_{16}O_{10}$	1.862
M0221	gallic acid 3-O-β-D-glucoside	$C_{13}H_{16}O_{10}$	1.163
M0222	Phlorin	$C_{12}H_{16}O_8$	2.366
M0223	vulgaxanthin i	$C_{14}H_{17}N_3O_7$	0.86
M0224	Tricrozarin A	$C_{13}H_{10}O_8$	1.821
M0225	[(2R,5R)-5-(2-Amino-6-oxo-1H-purin-9-yl)-3,4-dihydroxyoxolan-2-yl]methyl dihydrogen phosphate	$C_{10}H_{14}N_5O_8P$	1.184
M0226	picrocrocinicacido-β-d-glucopyrinoside	$C_{16}H_{26}O_8$	4.8
M0227	(1R*,3R*,3'S*)-1,2,3,4-Tetrahydro-1-(2-thio-3-pyrrolidinyl)-beta-carboline-3-carboxylic acid	$C_{16}H_{17}N_3O_2S$	2.367
M0228	11-methoxygelsemamide	$C_{21}H_{25}NO_4$	5.066
M0229	8-hydroxy-10-hydrosweroside	$C_{16}H_{24}O_{10}$	4.44
M0230	1- hydroxy- 7- hydroxymethyl- 1,4a,5,7a- tetrahydrocyclopenta［c］pyran- 4- carb Aldehyde	$C_{16}H_{24}O_9$	4.525

M0231	acetic acid	$C_{20}H_{19}C_{l}N_{2}O_{3}$	0.955
M0232	(z)-8-β-d-glucopyranosyloxycinnamicacid	$C_{15}H_{18}O_{8}$	4.7
M0233	Dioctyl phthalate	$C_{24}H_{38}O_{4}$	15.005
M0234	meliaionoside a	$C_{19}H_{36}O_{8}$	5.128
M0235	Epijasminoside A	$C_{16}H_{26}O_{7}$	4.983
M0236	(1r,2r)-p-menth-3-ene-1,2-diol 2-o-β-d-gluco-pyranoside	$C_{16}H_{28}O_{7}$	5.299
M0237	Dihydrosyringin	$C_{17}H_{26}O_{9}$	4.428
M0238	1-(4-hydroxy-3-methoxyphenyl-2-[4-(omega-hydroxypropyl)-2-methoxyphenoxy]-propane-1,3-diol	$C_{20}H_{26}O_{7}$	5.076
M0239	7-o-methylaloeresin	$C_{20}H_{24}O_{9}$	4.998
M0240	caesaldekarin e	$C_{24}H_{30}O_{6}$	9.957
M0241	Hexadecyl ferulate	$C_{26}H_{42}O_{4}$	11.835
M0242	Celacinnine	$C_{25}H_{31}N_{3}O_{2}$	5.178
M0243	ducheside b	$C_{19}H_{14}O_{12}$	4.651
M0244	Rehmaionoside C	$C_{19}H_{32}O_{8}$	4.998
M0245	(e)-2-hexenyl-α-l-arabinopyranosyl-(1→2)-β-d-glucopyranoside	$C_{17}H_{30}O_{10}$	4.721
M0246	massonivesinol	$C_{20}H_{30}O_{8}$	4.057
M0247	benzyl alcoholβ-d-(2'-o-β-xylopyranosyl)glucopyranoside	$C_{18}H_{26}O_{10}$	4.355
M0248	δ-tocopherol	$C_{27}H_{46}O_{2}$	10.291
M0249	staphylionoside c	$C_{19}H_{32}O_{9}$	4.861
M0250	(3s,5r,6r,7 e,9s)-megastigman-7-ene-3,5,6,9-tetrol-9-o-β-d-glucopyranoside	$C_{19}H_{34}O_{9}$	4.144
M0251	10-methoxy-20-o-acetylcamptothecin	$C_{23}H_{20}N_{2}O_{6}$	0.784
M0252	Sayaendoside	$C_{19}H_{28}O_{10}$	4.656
M0253	2,3-HHDP-D-glucopyranose	$C_{20}H_{18}O_{14}$	4.399
M0254	2,3-o-(s)-hexahydroxydiphenoyl-d-gluco-pyranose	$C_{20}H_{18}O_{14}$	1.3
M0255	Gallic acid 3-O-(6-galloylglucoside)	$C_{20}H_{20}O_{14}$	4.036
M0256	Gein	$C_{21}H_{30}O_{11}$	5.161

M0257	2',3'-dihydroxy-1'-propoxypseudolarate b	$C_{26}H_{34}O_{10}$	6.534
M0258	11-oxo-kansenonol	$C_{30}H_{46}O_4$	10.291
M0259	daturametelin f	$C_{27}H_{36}O_8S$	4.144
M0260	Eriojaposide A	$C_{24}H_{38}O_{11}$	4.943
M0261	Glyuranolide	$C_{31}H_{44}O_6$	8.939
M0262	cuneataside e	$C_{24}H_{40}O_{11}$	5.019
M0263	yemuoside ym1	$C_{25}H_{30}O_{11}$	4.407
M0264	(+)-Lariciresinol-4-beta-D-glucopyranoside	$C_{26}H_{34}O_{11}$	4.861
M0265	MGMG 18:2	$C_{27}H_{48}O_9$	11.134
M0266	(6s,9r)-vomifoliol-9-o-β-xylopyranosyl-(1''→6')-o-β-glucopyranoside	$C_{24}H_{38}O_{12}$	4.464
M0267	Icariside E5	$C_{26}H_{34}O_{11}$	4.861
M0268	(+) isolariciresinol 9-o-β-d-glucopyranoside	$C_{26}H_{34}O_{11}$	4.874
M0269	staphylionoside k	$C_{25}H_{44}O_{12}$	4.55
M0270	ah21	$C_{34}H_{28}O_8$	0.995
M0271	JusticidinosideA	$C_{28}H_{28}O_{13}$	0.784
M0272	Quercetin 3-galactosyl-(1->6)-glucoside	$C_{27}H_{30}O_{17}$	4.099
M0273	Amlaic acid	$C_{27}H_{24}O_{19}$	4.144
M0274	Madreselvin A	$C_{28}H_{32}O_{17}$	4.292
M0275	ajacine	$C_{34}H_{48}N_2O_9$	5.901
M0276	6''-o-p-hydroxybenzoyliridin	$C_{31}H_{30}O_{15}$	4.464
M0277	Gingerglycolipid A	$C_{33}H_{56}O_{14}$	9.99
M0278	evonine	$C_{36}H_{43}NO_{17}$	0.802
M0279	scropolioside D_qt	$C_{28}H_{32}O_{12}$	2.364
M0280	isoscoparin 2''-o-(6'''-(e)-coumaroyl)glucoside-4'-o-glucoside	$C_{43}H_{48}O_{23}$	4.677
M0281	calendasaponin a	$C_{54}H_{86}O_{24}$	8.099
M0282	esculentoside O	$C_{35}H_{54}O_{10}$	4.948

M0283	cimiracemoside e	$C_{37}H_{58}O_{11}$	5.011
M0284	Isolychnose	$C_{24}H_{42}O_{21}$	0.792
M0285	Myricitrin	$C_{15}H_{10}O_{8}$	4.747
M0286	Rutin	$C_{27}H_{30}O_{16}$	4.741
M0287	Taxifolin	$C_{15}H_{12}O_{7}$	2.355
M0288	Luteolin	$C_{15}H_{10}O_{6}$	7.028
M0289	Dihydromyricetin	$C_{15}H_{12}O_{8}$	6.203
M0290	Epicatechin	$C_{15}H_{14}O_{6}$	2.356
M0291	eriodictyol	$C_{15}H_{12}O_{6}$	0.835
M0292	Hederagenin	$C_{30}H_{48}O_{4}$	11.258
M0293	Di-O-Methylcrenatin	$C_{15}H_{22}O_{9}$	4.313

Table S2. List of 18 candidate bioactive compounds meeting the criteria of OB ⩾ 20% and DL ⩾ 0.18.

No.	Compounds	Formula	RT/min	OB	DL	Category
M0024	Kaempferol	$C_{15}H_{10}O_6$	7.028	41.88	0.24	Flavonoids
M0026	Ellagic acid	$C_{14}H_6O_8$	4.936	43.06	0.43	Phenols
M0027	Morin	$C_{15}H_{10}O_7$	6.203	46.23	0.7	Flavonoids
M0035	Kaempferol 7-rhamnoside	$C_{21}H_{20}O_{10}$	5.992	28.88	0.73	Flavonoids
M0037	Asperuloside acid	$C_{18}H_{24}O_{12}$	3.763	22.48	0.45	Terpenes
M0099	Norwogonin	$C_{15}H_{10}O_5$	7.215	39.4	0.21	Flavonoids
M0101	Kaurenoic acid	$C_{20}H_{30}O_2$	12.992	59.52	0.34	Terpenes
M0102	Chebulic acid	$C_{14}H_{12}O_{11}$	1.183	72	0.32	Phenols
M0108	Obacunone	$C_{26}H_{30}O_7$	9.966	43.29	0.77	Terpenes
M0110	Alisol B	$C_{30}H_{48}O_4$	12.288	36.76	0.82	Terpenes
M0114	Arjunolic acid	$C_{30}H_{48}O_5$	9.427	23.22	0.72	Terpenes
M0124	(+)-Syringaresinol O-beta-D-glucoside	$C_{28}H_{36}O_{13}$	5.096	43.35	0.77	Phenylpropanoids
M0287	Taxifolin	$C_{15}H_{12}O_7$	2.355	57.84	0.27	Flavonoids
M0288	Luteolin	$C_{15}H_{10}O_6$	7.028	36.16	0.25	Flavonoids
M0289	Dihydromyricetin	$C_{15}H_{12}O_8$	6.203	23.48	0.31	Flavonoids
M0290	Epicatechin	$C_{15}H_{14}O_6$	2.356	48.96	0.24	Flavonoids
M0291	eriodictyol	$C_{15}H_{12}O_6$	0.835	71.79	0.24	Flavonoids
M0292	Hederagenin	$C_{30}H_{48}O_4$	11.258	36.91	0.75	Terpenes

Table S3. Pearson correlation coefficients between DEGs and DEMs.

**DEMs**	**DEGs**	***r***	***p*-value**
3,4-Dihydroxymandelate	Apoa4	0.660302913	0.01943809
3,4-Dihydroxymandelate	Bco1	0.563176076	0.056566229
3,4-Dihydroxymandelate	Birc3	0.851903625	0.000435163
3,4-Dihydroxymandelate	C2	0.59054123	0.043208431
3,4-Dihydroxymandelate	Ccl5	0.420787923	0.173152766
3,4-Dihydroxymandelate	Ccl9	0.748835786	0.005070366
3,4-Dihydroxymandelate	Cd22	0.505937569	0.093304202
3,4-Dihydroxymandelate	Cd300a	0.605594544	0.036901124
3,4-Dihydroxymandelate	Ceacam20	0.663264051	0.018710906
3,4-Dihydroxymandelate	Cnr1	-0.71092431	0.009544957
3,4-Dihydroxymandelate	Creb3l3	0.540424795	0.069673456
3,4-Dihydroxymandelate	Fcnb	0.724171421	0.007738126
3,4-Dihydroxymandelate	Gale	0.600987995	0.038756469
3,4-Dihydroxymandelate	Gzmk	0.35898882	0.251800948
3,4-Dihydroxymandelate	Hmgcs2	0.606033089	0.036727862
3,4-Dihydroxymandelate	Igsf10	-0.454996109	0.137218942
3,4-Dihydroxymandelate	Incenp	0.381173888	0.221512245
3,4-Dihydroxymandelate	Itih1	0.622029542	0.030796741
3,4-Dihydroxymandelate	Klra5	0.718698475	0.008450389
3,4-Dihydroxymandelate	Lipg	-0.666084323	0.018037023
3,4-Dihydroxymandelate	LOC102546966	-0.684811802	0.014004127
3,4-Dihydroxymandelate	LOC102549836	0.676398278	0.015723354
3,4-Dihydroxymandelate	Matk	0.579176741	0.048449933
3,4-Dihydroxymandelate	Mlana	-0.812385941	0.00132348
3,4-Dihydroxymandelate	Mnda	0.846566496	0.000514558
3,4-Dihydroxymandelate	Mogat2	0.646495615	0.023104198
3,4-Dihydroxymandelate	Morn5	0.688990742	0.013203793
3,4-Dihydroxymandelate	Mttp	0.532372812	0.074773997
3,4-Dihydroxymandelate	Phospho1	0.742774948	0.005648942
3,4-Dihydroxymandelate	Pik3c2g	0.61416198	0.033620417
3,4-Dihydroxymandelate	Plin2	0.865273218	0.000277602
3,4-Dihydroxymandelate	Prc1	0.593233065	0.04202832
3,4-Dihydroxymandelate	Ptchd3	0.863659071	0.000293801
3,4-Dihydroxymandelate	S1pr4	0.592871981	0.042185278
3,4-Dihydroxymandelate	Sult1c3	0.872552368	0.000212988
3,4-Dihydroxymandelate	Tf	0.709554987	0.00974819
4-Aminobutanoate(GABA)	Apoa4	-0.156423441	0.627337039
4-Aminobutanoate(GABA)	Bco1	-0.166356901	0.605341165
4-Aminobutanoate(GABA)	Birc3	-0.33124729	0.29290653
4-Aminobutanoate(GABA)	C2	-0.222746304	0.486517424
4-Aminobutanoate(GABA)	Ccl5	0.03401089	0.916430282

4-Aminobutanoate(GABA)	Ccl9	-0.180747939	0.574005512
4-Aminobutanoate(GABA)	Cd22	-0.105086697	0.745158433
4-Aminobutanoate(GABA)	Cd300a	-0.334846461	0.287372195
4-Aminobutanoate(GABA)	Ceacam20	-0.411861728	0.183411809
4-Aminobutanoate(GABA)	Cnr1	0.378093313	0.225580241
4-Aminobutanoate(GABA)	Creb3l3	-0.107273112	0.740016387
4-Aminobutanoate(GABA)	Fcnb	0.001071785	0.997362408
4-Aminobutanoate(GABA)	Gale	-0.190820914	0.552466439
4-Aminobutanoate(GABA)	Gzmk	-0.044674455	0.890350995
4-Aminobutanoate(GABA)	Hmgcs2	-0.092210783	0.77562809
4-Aminobutanoate(GABA)	Igsf10	0.906059294	4.91225E-05
4-Aminobutanoate(GABA)	Incenp	-0.130302164	0.686483776
4-Aminobutanoate(GABA)	Itih1	-0.069993394	0.828870833
4-Aminobutanoate(GABA)	Klra5	-0.176898338	0.58232422
4-Aminobutanoate(GABA)	Lipg	0.579027574	0.048521568
4-Aminobutanoate(GABA)	LOC102546966	0.111169492	0.730877107
4-Aminobutanoate(GABA)	LOC102549836	-0.022485735	0.944701298
4-Aminobutanoate(GABA)	Matk	-0.272764299	0.391029887
4-Aminobutanoate(GABA)	Mlana	0.109542396	0.734689762
4-Aminobutanoate(GABA)	Mnda	-0.110319685	0.732867705
4-Aminobutanoate(GABA)	Mogat2	-0.158425408	0.622880854
4-Aminobutanoate(GABA)	Morn5	-0.162591958	0.613643802
4-Aminobutanoate(GABA)	Mttp	-0.010216644	0.974860976
4-Aminobutanoate(GABA)	Phospho1	-0.308162422	0.329812574
4-Aminobutanoate(GABA)	Pik3c2g	-0.169868227	0.597636262
4-Aminobutanoate(GABA)	Plin2	-0.10147569	0.753671716
4-Aminobutanoate(GABA)	Prc1	-0.077725925	0.81025377
4-Aminobutanoate(GABA)	Ptchd3	-0.168971487	0.599600412
4-Aminobutanoate(GABA)	S1pr4	-0.062386125	0.847265575
4-Aminobutanoate(GABA)	Sult1c3	-0.28666641	0.36633332
4-Aminobutanoate(GABA)	Tf	-0.341691696	0.277011552
4-Fumarylacetoacetate	Apoa4	0.451529213	0.140619374
4-Fumarylacetoacetate	Bco1	0.948061182	2.72734E-06
4-Fumarylacetoacetate	Birc3	0.664028193	0.01852653
4-Fumarylacetoacetate	C2	0.851517634	0.000440563
4-Fumarylacetoacetate	Ccl5	0.809220894	0.001430795
4-Fumarylacetoacetate	Ccl9	0.569068495	0.053474654
4-Fumarylacetoacetate	Cd22	0.950188413	2.22076E-06
4-Fumarylacetoacetate	Cd300a	0.953388712	1.60201E-06
4-Fumarylacetoacetate	Ceacam20	0.770272247	0.003375788
4-Fumarylacetoacetate	Cnr1	-0.670350148	0.017051771
4-Fumarylacetoacetate	Creb3l3	0.792964349	0.002091361
4-Fumarylacetoacetate	Fcnb	0.706679795	0.010185393
4-Fumarylacetoacetate	Gale	0.837114921	0.000682282

4-Fumarylacetoacetate	Gzmk	0.816498082	0.001193421
4-Fumarylacetoacetate	Hmgcs2	0.922812769	1.89353E-05
4-Fumarylacetoacetate	Igsf10	-0.201892839	0.529185225
4-Fumarylacetoacetate	Incenp	0.851080427	0.000446742
4-Fumarylacetoacetate	Itih1	0.646394148	0.023132869
4-Fumarylacetoacetate	Klra5	0.699757022	0.011297833
4-Fumarylacetoacetate	Lipg	-0.717472136	0.008616487
4-Fumarylacetoacetate	LOC102546966	-0.76032069	0.004097891
4-Fumarylacetoacetate	LOC102549836	0.844455111	0.000548894
4-Fumarylacetoacetate	Matk	0.900878974	6.3672E-05
4-Fumarylacetoacetate	Mlana	-0.846194008	0.000520491
4-Fumarylacetoacetate	Mnda	0.728213918	0.007241629
4-Fumarylacetoacetate	Mogat2	0.860361826	0.000329173
4-Fumarylacetoacetate	Morn5	0.875407515	0.000191129
4-Fumarylacetoacetate	Mttp	0.810944716	0.00137155
4-Fumarylacetoacetate	Phospho1	0.759929167	0.004128509
4-Fumarylacetoacetate	Pik3c2g	0.890270607	0.00010393
4-Fumarylacetoacetate	Plin2	0.827639937	0.000890059
4-Fumarylacetoacetate	Prc1	0.416826987	0.177659616
4-Fumarylacetoacetate	Ptchd3	0.748074392	0.005140478
4-Fumarylacetoacetate	S1pr4	0.862414896	0.000306782
4-Fumarylacetoacetate	Sult1c3	0.739863905	0.005943883
4-Fumarylacetoacetate	Tf	0.849331265	0.000472141
5-L-Glutamyl-taurine	Apoa4	-0.375793439	0.228646289
5-L-Glutamyl-taurine	Bco1	-0.646803037	0.023017489
5-L-Glutamyl-taurine	Birc3	-0.690037497	0.013008735
5-L-Glutamyl-taurine	C2	-0.544958036	0.066909893
5-L-Glutamyl-taurine	Ccl5	-0.451726864	0.140424065
5-L-Glutamyl-taurine	Ccl9	-0.470365078	0.12278779
5-L-Glutamyl-taurine	Cd22	-0.551261747	0.063194127
5-L-Glutamyl-taurine	Cd300a	-0.725284853	0.007598908
5-L-Glutamyl-taurine	Ceacam20	-0.72802561	0.007264212
5-L-Glutamyl-taurine	Cnr1	0.488748072	0.106880817
5-L-Glutamyl-taurine	Creb3l3	-0.550848565	0.063433196
5-L-Glutamyl-taurine	Fcnb	-0.464359556	0.128302629
5-L-Glutamyl-taurine	Gale	-0.58659979	0.04497842
5-L-Glutamyl-taurine	Gzmk	-0.459406986	0.132970199
5-L-Glutamyl-taurine	Hmgcs2	-0.576732912	0.049632942
5-L-Glutamyl-taurine	Igsf10	0.596458958	0.040644399
5-L-Glutamyl-taurine	Incenp	-0.482832346	0.111841091
5-L-Glutamyl-taurine	Itih1	-0.472084164	0.121238262
5-L-Glutamyl-taurine	Klra5	-0.5068411	0.09262448
5-L-Glutamyl-taurine	Lipg	0.745717291	0.005362173
5-L-Glutamyl-taurine	LOC102546966	0.666506794	0.017937631

5-L-Glutamyl-taurine	LOC102549836	-0.602452554	0.03815956
5-L-Glutamyl-taurine	Matk	-0.67261744	0.016544489
5-L-Glutamyl-taurine	Mlana	0.685312843	0.013906327
5-L-Glutamyl-taurine	Mnda	-0.510139176	0.090171679
5-L-Glutamyl-taurine	Mogat2	-0.72180181	0.00804076
5-L-Glutamyl-taurine	Morn5	-0.70508755	0.010433699
5-L-Glutamyl-taurine	Mttp	-0.607136647	0.036294436
5-L-Glutamyl-taurine	Phospho1	-0.807609066	0.001487952
5-L-Glutamyl-taurine	Pik3c2g	-0.614712743	0.033416933
5-L-Glutamyl-taurine	Plin2	-0.683787122	0.014205717
5-L-Glutamyl-taurine	Prc1	-0.303263525	0.337954825
5-L-Glutamyl-taurine	Ptchd3	-0.70677106	0.010171295
5-L-Glutamyl-taurine	S1pr4	-0.496704182	0.100443527
5-L-Glutamyl-taurine	Sult1c3	-0.74335745	0.005591274
5-L-Glutamyl-taurine	Tf	-0.730661704	0.006952845
Adenosine	Apoa4	0.488174978	0.107354829
Adenosine	Bco1	0.919594272	2.30982E-05
Adenosine	Birc3	0.636479738	0.026061089
Adenosine	C2	0.835241976	0.000720021
Adenosine	Ccl5	0.744845221	0.005445995
Adenosine	Ccl9	0.554246839	0.061485462
Adenosine	Cd22	0.946785476	3.07248E-06
Adenosine	Cd300a	0.929003243	1.25993E-05
Adenosine	Ceacam20	0.640918955	0.024718651
Adenosine	Cnr1	-0.608931533	0.035597307
Adenosine	Creb3l3	0.828447641	0.000870652
Adenosine	Fcnb	0.738933462	0.006040554
Adenosine	Gale	0.771842444	0.00327134
Adenosine	Gzmk	0.790105161	0.002228263
Adenosine	Hmgcs2	0.93174834	1.03935E-05
Adenosine	Igsf10	-0.068094927	0.833454383
Adenosine	Incenp	0.783542396	0.002568367
Adenosine	Itih1	0.650756267	0.021923445
Adenosine	Klra5	0.694697464	0.012166141
Adenosine	Lipg	-0.679794401	0.015011701
Adenosine	LOC102546966	-0.697258442	0.011720674
Adenosine	LOC102549836	0.789677574	0.002249311
Adenosine	Matk	0.86018771	0.000331129
Adenosine	Mlana	-0.739874982	0.005942739
Adenosine	Mnda	0.764032214	0.003816115
Adenosine	Mogat2	0.866111579	0.000269468
Adenosine	Morn5	0.829797572	0.000838946
Adenosine	Mttp	0.822690182	0.001016398
Adenosine	Phospho1	0.663543528	0.018643317

Adenosine	Pik3c2g	0.908555004	4.31205E-05
Adenosine	Plin2	0.829327908	0.000849874
Adenosine	Prc1	0.446169197	0.145982818
Adenosine	Ptchd3	0.759720425	0.004144904
Adenosine	S1pr4	0.847336741	0.000502455
Adenosine	Sult1c3	0.706799912	0.010166841
Adenosine	Tf	0.83260002	0.000775977
Carbamoyl phosphate	Apoa4	-0.079224695	0.806655415
Carbamoyl phosphate	Bco1	-0.259613318	0.415149777
Carbamoyl phosphate	Birc3	-0.069198751	0.830788786
Carbamoyl phosphate	C2	-0.312417992	0.322827149
Carbamoyl phosphate	Ccl5	0.047927106	0.882414871
Carbamoyl phosphate	Ccl9	-0.100000064	0.757157958
Carbamoyl phosphate	Cd22	-0.170355138	0.5965708
Carbamoyl phosphate	Cd300a	-0.354946446	0.257567897
Carbamoyl phosphate	Ceacam20	-0.255448033	0.422939683
Carbamoyl phosphate	Cnr1	0.372680865	0.232835248
Carbamoyl phosphate	Creb3l3	-0.216270803	0.499596635
Carbamoyl phosphate	Fcnb	0.075089768	0.816590997
Carbamoyl phosphate	Gale	-0.161889338	0.615197916
Carbamoyl phosphate	Gzmk	-0.037241983	0.908519083
Carbamoyl phosphate	Hmgcs2	-0.186893462	0.560824734
Carbamoyl phosphate	Igsf10	0.827904313	0.00088367
Carbamoyl phosphate	Incenp	-0.187939489	0.558593589
Carbamoyl phosphate	Itih1	-0.030712076	0.924514474
Carbamoyl phosphate	Klra5	-0.142996294	0.657514513
Carbamoyl phosphate	Lipg	0.59273878	0.042243283
Carbamoyl phosphate	LOC102546966	-0.065854578	0.838869475
Carbamoyl phosphate	LOC102549836	0.049761259	0.87794406
Carbamoyl phosphate	Matk	-0.430975572	0.161893241
Carbamoyl phosphate	Mlana	0.0317306	0.922017708
Carbamoyl phosphate	Mnda	-0.037610362	0.907617594
Carbamoyl phosphate	Mogat2	-0.153664587	0.633496729
Carbamoyl phosphate	Morn5	-0.077447433	0.810922768
Carbamoyl phosphate	Mttp	-0.038886994	0.904494229
Carbamoyl phosphate	Phospho1	-0.061438343	0.84956245
Carbamoyl phosphate	Pik3c2g	-0.223857553	0.484288706
Carbamoyl phosphate	Plin2	0.046804761	0.885152187
Carbamoyl phosphate	Prc1	0.015365001	0.962199592
Carbamoyl phosphate	Ptchd3	0.04958266	0.878379261
Carbamoyl phosphate	S1pr4	-0.096422743	0.76562671
Carbamoyl phosphate	Sult1c3	-0.242379801	0.447839169
Carbamoyl phosphate	Tf	-0.360571827	0.249563389
D-Galactose	Apoa4	0.63613376	0.026167883

D-Galactose	Bco1	0.812972707	0.001304284
D-Galactose	Birc3	0.729523834	0.007085992
D-Galactose	C2	0.822440903	0.001023107
D-Galactose	Ccl5	0.583823491	0.046255523
D-Galactose	Ccl9	0.703491114	0.010687152
D-Galactose	Cd22	0.789001197	0.002282915
D-Galactose	Cd300a	0.867872505	0.000252988
D-Galactose	Ceacam20	0.736060306	0.006346546
D-Galactose	Cnr1	-0.566826462	0.054636682
D-Galactose	Creb3l3	0.718379598	0.008493346
D-Galactose	Fcnb	0.768796607	0.003476237
D-Galactose	Gale	0.826059537	0.000928998
D-Galactose	Gzmk	0.579141026	0.048467078
D-Galactose	Hmgcs2	0.803930793	0.001624951
D-Galactose	Igsf10	-0.312728982	0.32231987
D-Galactose	Incenp	0.664046443	0.018522143
D-Galactose	Itih1	0.747509461	0.00519297
D-Galactose	Klra5	0.792690678	0.002104179
D-Galactose	Lipg	-0.69817115	0.011564877
D-Galactose	LOC102546966	-0.837877983	0.000667353
D-Galactose	LOC102549836	0.802254908	0.001690486
D-Galactose	Matk	0.884584667	0.000132478
D-Galactose	Mlana	-0.85299149	0.000420219
D-Galactose	Mnda	0.713570563	0.009161174
D-Galactose	Mogat2	0.797721468	0.001877947
D-Galactose	Morn5	0.811686559	0.001346642
D-Galactose	Mttp	0.813080873	0.001300769
D-Galactose	Phospho1	0.773171282	0.003184882
D-Galactose	Pik3c2g	0.800557501	0.00175891
D-Galactose	Plin2	0.841535017	0.00059928
D-Galactose	Prc1	0.586153509	0.045182009
D-Galactose	Ptchd3	0.871245208	0.000223625
D-Galactose	S1pr4	0.824276148	0.000974503
D-Galactose	Sult1c3	0.816773975	0.001185062
D-Galactose	Tf	0.920606067	2.17188E-05
D-Sorbitol	Apoa4	-0.553089174	0.062144255
D-Sorbitol	Bco1	-0.746835463	0.005256124
D-Sorbitol	Birc3	-0.801262116	0.001730254
D-Sorbitol	C2	-0.745990884	0.005336077
D-Sorbitol	Ccl5	-0.563798312	0.056234013
D-Sorbitol	Ccl9	-0.662890854	0.018801439
D-Sorbitol	Cd22	-0.705930831	0.010301638
D-Sorbitol	Cd300a	-0.834675954	0.000731737
D-Sorbitol	Ceacam20	-0.813521467	0.001286527

D-Sorbitol	Cnr1	0.705667456	0.010342749
D-Sorbitol	Creb3l3	-0.624681034	0.029884778
D-Sorbitol	Fcnb	-0.661279482	0.019196025
D-Sorbitol	Gale	-0.761674316	0.003993359
D-Sorbitol	Gzmk	-0.557306613	0.059767602
D-Sorbitol	Hmgcs2	-0.719057783	0.00840218
D-Sorbitol	Igsf10	0.587462886	0.044586525
D-Sorbitol	Incenp	-0.636178503	0.026154054
D-Sorbitol	Itih1	-0.639386891	0.025176148
D-Sorbitol	Klra5	-0.718090083	0.008532489
D-Sorbitol	Lipg	0.829952423	0.000835367
D-Sorbitol	LOC102546966	0.742851409	0.005641347
D-Sorbitol	LOC102549836	-0.753235434	0.004679264
D-Sorbitol	Matk	-0.79011497	0.002227782
D-Sorbitol	Mlana	0.795512935	0.001974842
D-Sorbitol	Mnda	-0.695220147	0.012074221
D-Sorbitol	Mogat2	-0.748404012	0.005110036
D-Sorbitol	Morn5	-0.793734566	0.002055608
D-Sorbitol	Mttp	-0.674863652	0.016052946
D-Sorbitol	Phospho1	-0.807691041	0.001485004
D-Sorbitol	Pik3c2g	-0.720863289	0.008163038
D-Sorbitol	Plin2	-0.810019048	0.001403124
D-Sorbitol	Prc1	-0.490798437	0.105196336
D-Sorbitol	Ptchd3	-0.800629494	0.001755966
D-Sorbitol	S1pr4	-0.687326376	0.01351838
D-Sorbitol	Sult1c3	-0.807781891	0.001481741
D-Sorbitol	Tf	-0.853810265	0.000409237
Deoxyguanosine	Apoa4	0.220906257	0.490218035
Deoxyguanosine	Bco1	0.818548684	0.001132362
Deoxyguanosine	Birc3	0.354913277	0.257615532
Deoxyguanosine	C2	0.784391907	0.002522246
Deoxyguanosine	Ccl5	0.797108594	0.001904462
Deoxyguanosine	Ccl9	0.365096621	0.243232264
Deoxyguanosine	Cd22	0.904725124	5.25899E-05
Deoxyguanosine	Cd300a	0.779267131	0.002810322
Deoxyguanosine	Ceacam20	0.605135653	0.037083047
Deoxyguanosine	Cnr1	-0.563805209	0.056230338
Deoxyguanosine	Creb3l3	0.570806947	0.052585618
Deoxyguanosine	Fcnb	0.503906044	0.094844747
Deoxyguanosine	Gale	0.746059479	0.00532955
Deoxyguanosine	Gzmk	0.785883504	0.002442798
Deoxyguanosine	Hmgcs2	0.758142722	0.004270417
Deoxyguanosine	Igsf10	-0.011958073	0.970577539
Deoxyguanosine	Incenp	0.90937251	4.12854E-05

Deoxyguanosine	Itih1	0.4812824	0.113165445
Deoxyguanosine	Klra5	0.511387699	0.089254701
Deoxyguanosine	Lipg	-0.52103745	0.082379315
Deoxyguanosine	LOC102546966	-0.46254288	0.130002157
Deoxyguanosine	LOC102549836	0.696295705	0.011886692
Deoxyguanosine	Matk	0.778779552	0.002838981
Deoxyguanosine	Mlana	-0.582499229	0.046873607
Deoxyguanosine	Mnda	0.501619502	0.096599019
Deoxyguanosine	Mogat2	0.566528261	0.054792557
Deoxyguanosine	Morn5	0.609108139	0.035529234
Deoxyguanosine	Mttp	0.578062058	0.04898704
Deoxyguanosine	Phospho1	0.427925469	0.165214214
Deoxyguanosine	Pik3c2g	0.675821364	0.015846668
Deoxyguanosine	Plin2	0.510518913	0.089892111
Deoxyguanosine	Prc1	0.233785287	0.464586466
Deoxyguanosine	Ptchd3	0.341545463	0.277230617
Deoxyguanosine	S1pr4	0.800523941	0.001760284
Deoxyguanosine	Sult1c3	0.345681407	0.271073026
Deoxyguanosine	Tf	0.624865329	0.029822122
Dopamine	Apoa4	-0.548541953	0.06477933
Dopamine	Bco1	-0.746557542	0.005282333
Dopamine	Birc3	-0.774052278	0.003128528
Dopamine	C2	-0.769669599	0.003416541
Dopamine	Ccl5	-0.562760606	0.056788813
Dopamine	Ccl9	-0.643818089	0.023869469
Dopamine	Cd22	-0.705959504	0.010297169
Dopamine	Cd300a	-0.827890276	0.000884009
Dopamine	Ceacam20	-0.777194607	0.002933681
Dopamine	Cnr1	0.681415979	0.014680406
Dopamine	Creb3l3	-0.622825231	0.030520996
Dopamine	Fcnb	-0.648604753	0.022514061
Dopamine	Gale	-0.750940118	0.004880344
Dopamine	Gzmk	-0.559860931	0.058359314
Dopamine	Hmgcs2	-0.71110668	0.00951813
Dopamine	Igsf10	0.569670034	0.053165849
Dopamine	Incenp	-0.632286688	0.027376809
Dopamine	Itih1	-0.641515297	0.024542215
Dopamine	Klra5	-0.71701188	0.00867945
Dopamine	Lipg	0.796544623	0.001929114
Dopamine	LOC102546966	0.734625556	0.006503639
Dopamine	LOC102549836	-0.753618892	0.004646291
Dopamine	Matk	-0.80505371	0.001582143
Dopamine	Mlana	0.79093807	0.002187696
Dopamine	Mnda	-0.645268392	0.023452705

Dopamine	Mogat2	-0.738194066	0.006118215
Dopamine	Morn5	-0.764836981	0.003757008
Dopamine	Mttp	-0.693870369	0.012312654
Dopamine	Phospho1	-0.778674631	0.002845177
Dopamine	Pik3c2g	-0.716369229	0.008767939
Dopamine	Plin2	-0.785771128	0.002448716
Dopamine	Prc1	-0.491078644	0.104967513
Dopamine	Ptchd3	-0.791197097	0.002175195
Dopamine	S1pr4	-0.710096028	0.009667508
Dopamine	Sult1c3	-0.785737574	0.002450485
Dopamine	Tf	-0.863291577	0.00029759
FAICAR	Apoa4	0.48525231	0.10979393
FAICAR	Bco1	0.692816792	0.012501174
FAICAR	Birc3	0.211856958	0.508600535
FAICAR	C2	0.775268783	0.003051963
FAICAR	Ccl5	0.473662296	0.119827135
FAICAR	Ccl9	0.435408703	0.157142365
FAICAR	Cd22	0.744258895	0.005502905
FAICAR	Cd300a	0.69689468	0.011783199
FAICAR	Ceacam20	0.261157274	0.412280511
FAICAR	Cnr1	-0.261923897	0.410859511
FAICAR	Creb3l3	0.709479418	0.009759498
FAICAR	Fcnb	0.652772141	0.021380378
FAICAR	Gale	0.647871933	0.022717846
FAICAR	Gzmk	0.536350317	0.072223517
FAICAR	Hmgcs2	0.802986644	0.001661627
FAICAR	Igsf10	0.311039697	0.325080647
FAICAR	Incenp	0.598705545	0.039699929
FAICAR	Itih1	0.610023272	0.035177988
FAICAR	Klra5	0.665461239	0.018184349
FAICAR	Lipg	-0.256632656	0.420716922
FAICAR	LOC102546966	-0.637148465	0.025855564
FAICAR	LOC102549836	0.600633106	0.038902105
FAICAR	Matk	0.783569232	0.002566901
FAICAR	Mlana	-0.554040271	0.061602657
FAICAR	Mnda	0.427964137	0.165171845
FAICAR	Mogat2	0.597169687	0.0403439
FAICAR	Morn5	0.577674928	0.049174553
FAICAR	Mttp	0.79280806	0.002098674
FAICAR	Phospho1	0.250811634	0.43169444
FAICAR	Pik3c2g	0.738805616	0.006053929
FAICAR	Plin2	0.519176772	0.083676036
FAICAR	Prc1	0.476436029	0.117373179
FAICAR	Ptchd3	0.562410397	0.056976907

FAICAR	S1pr4	0.737795795	0.006160357
FAICAR	Sult1c3	0.456936495	0.135339202
FAICAR	Tf	0.689486214	0.013111196
GMP	Apoa4	0.544852123	0.066973582
GMP	Bco1	0.902011237	6.02355E-05
GMP	Birc3	0.709408765	0.00977008
GMP	C2	0.829885713	0.000836908
GMP	Ccl5	0.723592119	0.00781131
GMP	Ccl9	0.622979234	0.030467833
GMP	Cd22	0.90031978	6.5425E-05
GMP	Cd300a	0.909193434	4.1682E-05
GMP	Ceacam20	0.706073495	0.010279419
GMP	Cnr1	-0.630018768	0.028108111
GMP	Creb3l3	0.81087936	0.001373761
GMP	Fcnb	0.768963359	0.003464774
GMP	Gale	0.78759464	0.002354024
GMP	Gzmk	0.735558056	0.00640121
GMP	Hmgcs2	0.910271413	3.93391E-05
GMP	Igsf10	-0.145575056	0.651680544
GMP	Incenp	0.743418844	0.005585221
GMP	Itih1	0.675909224	0.015827842
GMP	Klra5	0.735054477	0.006456373
GMP	Lipg	-0.673111769	0.016435377
GMP	LOC102546966	-0.782732076	0.002612957
GMP	LOC102549836	0.812584683	0.001316954
GMP	Matk	0.868234826	0.000249697
GMP	Mlana	-0.853441352	0.000414157
GMP	Mnda	0.815068844	0.001237455
GMP	Mogat2	0.869978518	0.000234323
GMP	Morn5	0.844055514	0.000555588
GMP	Mttp	0.805438905	0.001567659
GMP	Phospho1	0.757642965	0.004310768
GMP	Pik3c2g	0.899946468	6.66163E-05
GMP	Plin2	0.879034155	0.000165933
GMP	Prc1	0.496067683	0.100948728
GMP	Ptchd3	0.840257831	0.000622411
GMP	S1pr4	0.859460923	0.000339391
GMP	Sult1c3	0.801962973	0.001702106
GMP	Tf	0.857353029	0.000364264
Guanosine	Apoa4	-0.513671313	0.08759384
Guanosine	Bco1	-0.61405989	0.033658232
Guanosine	Birc3	-0.49316237	0.103276292
Guanosine	C2	-0.52862554	0.077232651
Guanosine	Ccl5	-0.353382299	0.25981981

Guanosine	Ccl9	-0.533432373	0.074088643
Guanosine	Cd22	-0.463100131	0.12947929
Guanosine	Cd300a	-0.692733124	0.012516236
Guanosine	Ceacam20	-0.549519376	0.064206518
Guanosine	Cnr1	0.576051664	0.049966306
Guanosine	Creb3l3	-0.663042307	0.01876466
Guanosine	Fcnb	-0.505508221	0.093628371
Guanosine	Gale	-0.581835155	0.047185738
Guanosine	Gzmk	-0.391039218	0.208783913
Guanosine	Hmgcs2	-0.675716302	0.015869201
Guanosine	Igsf10	0.500426175	0.097523147
Guanosine	Incenp	-0.355541342	0.256714417
Guanosine	Itih1	-0.509136239	0.090912876
Guanosine	Klra5	-0.628529224	0.028596026
Guanosine	Lipg	0.575688231	0.050144791
Guanosine	LOC102546966	0.699637715	0.011317762
Guanosine	LOC102549836	-0.528735297	0.077159863
Guanosine	Matk	-0.639877279	0.025029049
Guanosine	Mlana	0.742788813	0.005647564
Guanosine	Mnda	-0.404756752	0.191841978
Guanosine	Mogat2	-0.661673355	0.019099021
Guanosine	Morn5	-0.781705254	0.002670306
Guanosine	Mttp	-0.693515528	0.01237591
Guanosine	Phospho1	-0.620242795	0.031422456
Guanosine	Pik3c2g	-0.675922105	0.015825084
Guanosine	Plin2	-0.613906737	0.033715019
Guanosine	Prc1	-0.452256336	0.13990173
Guanosine	Ptchd3	-0.708418934	0.009919233
Guanosine	S1pr4	-0.450245056	0.141892583
Guanosine	Sult1c3	-0.820791051	0.001068377
Guanosine	Tf	-0.674698769	0.016088658
Hydroxymethylbilane	Apoa4	-0.492159076	0.104088313
Hydroxymethylbilane	Bco1	-0.637565699	0.025727929
Hydroxymethylbilane	Birc3	-0.654251691	0.020988081
Hydroxymethylbilane	C2	-0.637733087	0.025676852
Hydroxymethylbilane	Ccl5	-0.668499878	0.017474135
Hydroxymethylbilane	Ccl9	-0.572853735	0.051552234
Hydroxymethylbilane	Cd22	-0.681379568	0.014687785
Hydroxymethylbilane	Cd300a	-0.636121187	0.02617177
Hydroxymethylbilane	Ceacam20	-0.59087238	0.043062003
Hydroxymethylbilane	Cnr1	0.369261487	0.237489427
Hydroxymethylbilane	Creb3l3	-0.530956896	0.075696612
Hydroxymethylbilane	Fcnb	-0.754364783	0.004582658
Hydroxymethylbilane	Gale	-0.719640554	0.008324427

Hydroxymethylbilane	Gzmk	-0.603466116	0.037750317
Hydroxymethylbilane	Hmgcs2	-0.669985769	0.017134349
Hydroxymethylbilane	Igsf10	-0.174334775	0.58788997
Hydroxymethylbilane	Incenp	-0.606884117	0.036393294
Hydroxymethylbilane	Itih1	-0.677045014	0.015585956
Hydroxymethylbilane	Klra5	-0.665009491	0.018291712
Hydroxymethylbilane	Lipg	0.334143077	0.288449073
Hydroxymethylbilane	LOC102546966	0.854118705	0.000405159
Hydroxymethylbilane	LOC102549836	-0.864484838	0.000285425
Hydroxymethylbilane	Matk	-0.599698798	0.03928738
Hydroxymethylbilane	Mlana	0.807109321	0.001506025
Hydroxymethylbilane	Mnda	-0.600495452	0.038958699
Hydroxymethylbilane	Mogat2	-0.667289782	0.017754483
Hydroxymethylbilane	Morn5	-0.754172235	0.004599021
Hydroxymethylbilane	Mttp	-0.769442631	0.003431986
Hydroxymethylbilane	Phospho1	-0.704445468	0.010535094
Hydroxymethylbilane	Pik3c2g	-0.617342746	0.032457409
Hydroxymethylbilane	Plin2	-0.823582262	0.000992666
Hydroxymethylbilane	Prc1	-0.515091047	0.086571887
Hydroxymethylbilane	Ptchd3	-0.822882612	0.001011242
Hydroxymethylbilane	S1pr4	-0.722074216	0.008005527
Hydroxymethylbilane	Sult1c3	-0.590131235	0.04339021
Hydroxymethylbilane	Tf	-0.656269731	0.020461503
L-Glutamate	Apoa4	-0.479974619	0.114290912
L-Glutamate	Bco1	-0.800410934	0.001764916
L-Glutamate	Birc3	-0.586250734	0.045137601
L-Glutamate	C2	-0.715033655	0.008953992
L-Glutamate	Ccl5	-0.673906376	0.016261096
L-Glutamate	Ccl9	-0.555676612	0.060678531
L-Glutamate	Cd22	-0.7803104	0.002749742
L-Glutamate	Cd300a	-0.866403234	0.000266683
L-Glutamate	Ceacam20	-0.660293823	0.019440354
L-Glutamate	Cnr1	0.618081407	0.032191512
L-Glutamate	Creb3l3	-0.745586067	0.005374724
L-Glutamate	Fcnb	-0.689824103	0.013048326
L-Glutamate	Gale	-0.786981293	0.002385556
L-Glutamate	Gzmk	-0.708526429	0.009902953
L-Glutamate	Hmgcs2	-0.869808504	0.000235788
L-Glutamate	Igsf10	0.171528503	0.594006226
L-Glutamate	Incenp	-0.70030697	0.011206306
L-Glutamate	Itih1	-0.6433157	0.024015083
L-Glutamate	Klra5	-0.711118181	0.00951644
L-Glutamate	Lipg	0.643928705	0.023837494
L-Glutamate	LOC102546966	0.796409442	0.001935059

L-Glutamate	LOC102549836	-0.806320686	0.001534887
L-Glutamate	Matk	-0.748557512	0.005095907
L-Glutamate	Mlana	0.755980062	0.004447116
L-Glutamate	Mnda	-0.554142723	0.061544512
L-Glutamate	Mogat2	-0.797669473	0.001880185
L-Glutamate	Morn5	-0.939267663	5.87321E-06
L-Glutamate	Mttp	-0.868108977	0.000250837
L-Glutamate	Phospho1	-0.669728707	0.017192783
L-Glutamate	Pik3c2g	-0.801568327	0.001717912
L-Glutamate	Plin2	-0.782835449	0.002607237
L-Glutamate	Prc1	-0.459729413	0.132663018
L-Glutamate	Ptchd3	-0.755091008	0.004521337
L-Glutamate	S1pr4	-0.647796069	0.022739019
L-Glutamate	Sult1c3	-0.710653752	0.00958486
L-Glutamate	Tf	-0.756189864	0.004429736
L-Histidine	Apoa4	-0.505259798	0.093816283
L-Histidine	Bco1	-0.761662953	0.003994228
L-Histidine	Birc3	-0.792981925	0.00209054
L-Histidine	C2	-0.771244306	0.003310833
L-Histidine	Ccl5	-0.568073942	0.053987967
L-Histidine	Ccl9	-0.598300813	0.039868915
L-Histidine	Cd22	-0.720174681	0.008253637
L-Histidine	Cd300a	-0.853790916	0.000409494
L-Histidine	Ceacam20	-0.753689029	0.004640279
L-Histidine	Cnr1	0.804844859	0.001590038
L-Histidine	Creb3l3	-0.666261256	0.017995348
L-Histidine	Fcnb	-0.609871929	0.035235904
L-Histidine	Gale	-0.682659802	0.014429965
L-Histidine	Gzmk	-0.600677675	0.038883794
L-Histidine	Hmgcs2	-0.750254949	0.004941614
L-Histidine	Igsf10	0.66498284	0.018298061
L-Histidine	Incenp	-0.631065348	0.027768907
L-Histidine	Itih1	-0.56535978	0.055406334
L-Histidine	Klra5	-0.6835699	0.014248725
L-Histidine	Lipg	0.898976932	6.97897E-05
L-Histidine	LOC102546966	0.633173252	0.027094704
L-Histidine	LOC102549836	-0.707627037	0.010039775
L-Histidine	Matk	-0.781981617	0.002654778
L-Histidine	Mlana	0.720456866	0.00821642
L-Histidine	Mnda	-0.698724757	0.011471129
L-Histidine	Mogat2	-0.767496262	0.003566621
L-Histidine	Morn5	-0.760700426	0.00406836
L-Histidine	Mttp	-0.642722897	0.02418773
L-Histidine	Phospho1	-0.735028499	0.006459228

L-Histidine	Pik3c2g	-0.758250101	0.004261784
L-Histidine	Plin2	-0.786515845	0.002409699
L-Histidine	Prc1	-0.426269727	0.167034882
L-Histidine	Ptchd3	-0.751054394	0.004870182
L-Histidine	S1pr4	-0.649963009	0.022139874
L-Histidine	Sult1c3	-0.79934193	0.0018092
L-Histidine	Tf	-0.838075864	0.000663523
Porphobilinogen	Apoa4	-0.269925924	0.396174007
Porphobilinogen	Bco1	-0.757724788	0.004304142
Porphobilinogen	Birc3	-0.669187862	0.017316201
Porphobilinogen	C2	-0.609028026	0.035560102
Porphobilinogen	Ccl5	-0.570684742	0.052647772
Porphobilinogen	Ccl9	-0.425512551	0.167871678
Porphobilinogen	Cd22	-0.731264414	0.006883085
Porphobilinogen	Cd300a	-0.820118476	0.001087269
Porphobilinogen	Ceacam20	-0.847968854	0.000492689
Porphobilinogen	Cnr1	0.535219787	0.072942273
Porphobilinogen	Creb3l3	-0.554684892	0.061237448
Porphobilinogen	Fcnb	-0.433960303	0.158684699
Porphobilinogen	Gale	-0.659431055	0.01965608
Porphobilinogen	Gzmk	-0.583515008	0.046398987
Porphobilinogen	Hmgcs2	-0.628974068	0.028449679
Porphobilinogen	Igsf10	0.5816248	0.047284915
Porphobilinogen	Incenp	-0.693650104	0.012351891
Porphobilinogen	Itih1	-0.417279781	0.177140742
Porphobilinogen	Klra5	-0.442852069	0.14936697
Porphobilinogen	Lipg	0.847867848	0.00049424
Porphobilinogen	LOC102546966	0.559356318	0.058635675
Porphobilinogen	LOC102549836	-0.574119124	0.050920538
Porphobilinogen	Matk	-0.752547042	0.0047389
Porphobilinogen	Mlana	0.616864173	0.032630521
Porphobilinogen	Mnda	-0.650105826	0.022100794
Porphobilinogen	Mogat2	-0.714747208	0.008994276
Porphobilinogen	Morn5	-0.663693599	0.018607098
Porphobilinogen	Mttp	-0.479289921	0.114883095
Porphobilinogen	Phospho1	-0.804814233	0.001591199
Porphobilinogen	Pik3c2g	-0.664520307	0.018408496
Porphobilinogen	Plin2	-0.64026769	0.024912386
Porphobilinogen	Prc1	-0.192095357	0.549765307
Porphobilinogen	Ptchd3	-0.561251713	0.057602323
Porphobilinogen	S1pr4	-0.596613251	0.040579028
Porphobilinogen	Sult1c3	-0.636738202	0.025981514
Porphobilinogen	Tf	-0.729867231	0.00704561
Pyridoxal 5'- phosphate	Apoa4	0.815772148	0.001215633

Pyridoxal 5'- phosphate	Bco1	0.67697712	0.015600338
Pyridoxal 5'- phosphate	Birc3	0.552397533	0.062540175
Pyridoxal 5'- phosphate	C2	0.706837504	0.01016104
Pyridoxal 5'- phosphate	Ccl5	0.315808997	0.317319465
Pyridoxal 5'- phosphate	Ccl9	0.753407042	0.004664486
Pyridoxal 5'- phosphate	Cd22	0.611497345	0.034617439
Pyridoxal 5'- phosphate	Cd300a	0.713690272	0.00914409
Pyridoxal 5'- phosphate	Ceacam20	0.351146024	0.263059202
Pyridoxal 5'- phosphate	Cnr1	-0.540098449	0.069875386
Pyridoxal 5'- phosphate	Creb3l3	0.797467434	0.001888903
Pyridoxal 5'- phosphate	Fcnb	0.80610185	0.001542971
Pyridoxal 5'- phosphate	Gale	0.64538961	0.023418113
Pyridoxal 5'- phosphate	Gzmk	0.365001422	0.243364481
Pyridoxal 5'- phosphate	Hmgcs2	0.785987535	0.002437329
Pyridoxal 5'- phosphate	Igsf10	-0.145293277	0.652317153
Pyridoxal 5'- phosphate	Incenp	0.338771343	0.281405158
Pyridoxal 5'- phosphate	Itih1	0.772619368	0.003220578
Pyridoxal 5'- phosphate	Klra5	0.834119414	0.000743401
Pyridoxal 5'- phosphate	Lipg	-0.545196278	0.066766783
Pyridoxal 5'- phosphate	LOC102546966	-0.644854827	0.023571005
Pyridoxal 5'- phosphate	LOC102549836	0.597535164	0.04018999
Pyridoxal 5'- phosphate	Matk	0.740984063	0.005829052
Pyridoxal 5'- phosphate	Mlana	-0.688217733	0.013349221
Pyridoxal 5'- phosphate	Mnda	0.676422	0.015718298
Pyridoxal 5'- phosphate	Mogat2	0.710678844	0.009581154
Pyridoxal 5'- phosphate	Morn5	0.721732852	0.008049697
Pyridoxal 5'- phosphate	Mttp	0.83298343	0.000767655
Pyridoxal 5'- phosphate	Phospho1	0.452147482	0.140009014
Pyridoxal 5'- phosphate	Pik3c2g	0.785679384	0.002453556
Pyridoxal 5'- phosphate	Plin2	0.765382341	0.003717349
Pyridoxal 5'- phosphate	Prc1	0.752743558	0.004721817
Pyridoxal 5'- phosphate	Ptchd3	0.851990282	0.000433957
Pyridoxal 5'- phosphate	S1pr4	0.718880131	0.00842599
Pyridoxal 5'- phosphate	Sult1c3	0.843735034	0.000561002
Pyridoxal 5'- phosphate	Tf	0.829142201	0.000854225
Pyridoxine 5'- phosphate	Apoa4	-0.525683675	0.079201146
Pyridoxine 5'- phosphate	Bco1	-0.756158258	0.004432351
Pyridoxine 5'- phosphate	Birc3	-0.692065816	0.012636847
Pyridoxine 5'- phosphate	C2	-0.856114489	0.000379525
Pyridoxine 5'- phosphate	Ccl5	-0.576424427	0.049783703
Pyridoxine 5'- phosphate	Ccl9	-0.600207078	0.039077448
Pyridoxine 5'- phosphate	Cd22	-0.833465649	0.000757286
Pyridoxine 5'- phosphate	Cd300a	-0.899760169	6.72171E-05
Pyridoxine 5'- phosphate	Ceacam20	-0.720503191	0.008210322

Pyridoxine 5'- phosphate	Cnr1	0.664892829	0.018319514
Pyridoxine 5'- phosphate	Creb3l3	-0.641913202	0.024424998
Pyridoxine 5'- phosphate	Fcnb	-0.702874736	0.010786224
Pyridoxine 5'- phosphate	Gale	-0.805403086	0.001569002
Pyridoxine 5'- phosphate	Gzmk	-0.642261786	0.024322645
Pyridoxine 5'- phosphate	Hmgcs2	-0.800642996	0.001755414
Pyridoxine 5'- phosphate	Igsf10	0.381222187	0.22144882
Pyridoxine 5'- phosphate	Incenp	-0.780698043	0.002727488
Pyridoxine 5'- phosphate	Itih1	-0.652048397	0.02157421
Pyridoxine 5'- phosphate	Klra5	-0.747470551	0.0051966
Pyridoxine 5'- phosphate	Lipg	0.843435095	0.000566106
Pyridoxine 5'- phosphate	LOC102546966	0.678438028	0.015293018
Pyridoxine 5'- phosphate	LOC102549836	-0.75511512	0.004519312
Pyridoxine 5'- phosphate	Matk	-0.824577973	0.000966684
Pyridoxine 5'- phosphate	Mlana	0.573412564	0.051272586
Pyridoxine 5'- phosphate	Mnda	-0.682785288	0.014404875
Pyridoxine 5'- phosphate	Mogat2	-0.713001178	0.009242762
Pyridoxine 5'- phosphate	Morn5	-0.7564511	0.004408167
Pyridoxine 5'- phosphate	Mttp	-0.698807203	0.011457216
Pyridoxine 5'- phosphate	Phospho1	-0.597103748	0.040371712
Pyridoxine 5'- phosphate	Pik3c2g	-0.753686032	0.004640536
Pyridoxine 5'- phosphate	Plin2	-0.742982921	0.005628301
Pyridoxine 5'- phosphate	Prc1	-0.474617461	0.118978317
Pyridoxine 5'- phosphate	Ptchd3	-0.694456534	0.012208686
Pyridoxine 5'- phosphate	S1pr4	-0.691757898	0.01269279
Pyridoxine 5'- phosphate	Sult1c3	-0.602219819	0.038253975
Pyridoxine 5'- phosphate	Tf	-0.857822653	0.000358604
SAICAR	Apoa4	-0.597539203	0.040188292
SAICAR	Bco1	-0.789465933	0.002259785
SAICAR	Birc3	-0.78466989	0.002507293
SAICAR	C2	-0.818437103	0.001135621
SAICAR	Ccl5	-0.605844966	0.036802115
SAICAR	Ccl9	-0.719509835	0.00834182
SAICAR	Cd22	-0.793716507	0.002056441
SAICAR	Cd300a	-0.874149505	0.000200533
SAICAR	Ceacam20	-0.818842244	0.001123822
SAICAR	Cnr1	0.781053551	0.002707199
SAICAR	Creb3l3	-0.643564569	0.023942869
SAICAR	Fcnb	-0.723084295	0.007875889
SAICAR	Gale	-0.834533277	0.000734714
SAICAR	Gzmk	-0.600412637	0.038992775
SAICAR	Hmgcs2	-0.773223808	0.003181501
SAICAR	Igsf10	0.534265337	0.07355289
SAICAR	Incenp	-0.726508492	0.007448083

SAICAR	Itih1	-0.69376324	0.012331726
SAICAR	Klra5	-0.775525548	0.003035987
SAICAR	Lipg	0.868152715	0.00025044
SAICAR	LOC102546966	0.692100007	0.012630646
SAICAR	LOC102549836	-0.776350416	0.002985092
SAICAR	Matk	-0.831272039	0.000805343
SAICAR	Mlana	0.76093249	0.004050392
SAICAR	Mnda	-0.769268962	0.003443839
SAICAR	Mogat2	-0.722384835	0.007965492
SAICAR	Morn5	-0.796859661	0.001915313
SAICAR	Mttp	-0.667251322	0.017763447
SAICAR	Phospho1	-0.732109971	0.006786104
SAICAR	Pik3c2g	-0.748895658	0.005064884
SAICAR	Plin2	-0.810989652	0.001370031
SAICAR	Prc1	-0.543129535	0.068015314
SAICAR	Ptchd3	-0.753604271	0.004647545
SAICAR	S1pr4	-0.764811872	0.003758841
SAICAR	Sult1c3	-0.762244864	0.00394991
SAICAR	Tf	-0.875763529	0.000188531
Sucrose	Apoa4	0.836870931	0.000687109
Sucrose	Bco1	0.720665154	0.00818903
Sucrose	Birc3	0.701304774	0.011041649
Sucrose	C2	0.852490845	0.000427046
Sucrose	Ccl5	0.49114244	0.104915462
Sucrose	Ccl9	0.871265878	0.000223453
Sucrose	Cd22	0.757106404	0.004354412
Sucrose	Cd300a	0.785091597	0.002484736
Sucrose	Ceacam20	0.595147913	0.041202878
Sucrose	Cnr1	-0.587642955	0.044505068
Sucrose	Creb3l3	0.67724914	0.015542774
Sucrose	Fcnb	0.935058413	8.15209E-06
Sucrose	Gale	0.882172761	0.000146294
Sucrose	Gzmk	0.471178136	0.122053317
Sucrose	Hmgcs2	0.791584255	0.002156614
Sucrose	Igsf10	-0.125074459	0.698530005
Sucrose	Incenp	0.607733874	0.036061402
Sucrose	Itih1	0.913692117	3.25825E-05
Sucrose	Klra5	0.943129394	4.25671E-06
Sucrose	Lipg	-0.634506635	0.026674386
Sucrose	LOC102546966	-0.787621758	0.002352637
Sucrose	LOC102549836	0.832949699	0.000768384
Sucrose	Matk	0.829643225	0.000842525
Sucrose	Mlana	-0.763861907	0.003828713
Sucrose	Mnda	0.765958234	0.003675817

Sucrose	Mogat2	0.681804144	0.014601908
Sucrose	Morn5	0.783511254	0.00257007
Sucrose	Mttp	0.847430133	0.000501003
Sucrose	Phospho1	0.571704089	0.052130898
Sucrose	Pik3c2g	0.735103147	0.006451026
Sucrose	Plin2	0.860305545	0.000329805
Sucrose	Prc1	0.812166459	0.001330715
Sucrose	Ptchd3	0.877668647	0.000175095
Sucrose	S1pr4	0.860071445	0.00033244
Sucrose	Sult1c3	0.75095643	0.004878893
Sucrose	Tf	0.919819891	2.27848E-05
Taurine	Apoa4	-0.385938878	0.215307505
Taurine	Bco1	-0.804409001	0.001606613
Taurine	Birc3	-0.797793033	0.001874869
Taurine	C2	-0.657980957	0.020022595
Taurine	Ccl5	-0.658236837	0.019957561
Taurine	Ccl9	-0.523360663	0.080779518
Taurine	Cd22	-0.747427727	0.005200598
Taurine	Cd300a	-0.847942501	0.000493093
Taurine	Ceacam20	-0.854290042	0.000402907
Taurine	Cnr1	0.66090607	0.019288322
Taurine	Creb3l3	-0.651236712	0.021793114
Taurine	Fcnb	-0.558348815	0.059190176
Taurine	Gale	-0.682717813	0.014418362
Taurine	Gzmk	-0.65668656	0.020353951
Taurine	Hmgcs2	-0.722410832	0.007962148
Taurine	Igsf10	0.543445349	0.067823495
Taurine	Incenp	-0.661955186	0.019029831
Taurine	Itih1	-0.500414359	0.097532328
Taurine	Klra5	-0.558953368	0.058857012
Taurine	Lipg	0.808470145	0.001457204
Taurine	LOC102546966	0.699618269	0.011321013
Taurine	LOC102549836	-0.701173033	0.011063285
Taurine	Matk	-0.748725327	0.005080493
Taurine	Mlana	0.806072989	0.001544039
Taurine	Mnda	-0.730870235	0.006928649
Taurine	Mogat2	-0.814954255	0.001241039
Taurine	Morn5	-0.791192985	0.002175393
Taurine	Mttp	-0.606722161	0.036456796
Taurine	Phospho1	-0.907564622	4.54289E-05
Taurine	Pik3c2g	-0.7593425	0.004174711
Taurine	Plin2	-0.80538429	0.001569707
Taurine	Prc1	-0.315480432	0.317850844
Taurine	Ptchd3	-0.759887107	0.004131808

Taurine	S1pr4	-0.668427531	0.017490804
Taurine	Sult1c3	-0.800678032	0.001753982
Taurine	Tf	-0.772679015	0.003216705
Tyrosine	Apoa4	-0.561679596	0.057370812
Tyrosine	Bco1	-0.763518812	0.003854189
Tyrosine	Birc3	-0.771190012	0.003314436
Tyrosine	C2	-0.780323532	0.002748986
Tyrosine	Ccl5	-0.577902069	0.049064472
Tyrosine	Ccl9	-0.653824997	0.021100674
Tyrosine	Cd22	-0.724188802	0.007735938
Tyrosine	Cd300a	-0.83677944	0.000688926
Tyrosine	Ceacam20	-0.772503733	0.003228095
Tyrosine	Cnr1	0.671670774	0.016754927
Tyrosine	Creb3l3	-0.64028861	0.024906145
Tyrosine	Fcnb	-0.667607503	0.017680558
Tyrosine	Gale	-0.764880939	0.003753799
Tyrosine	Gzmk	-0.573341497	0.051308091
Tyrosine	Hmgcs2	-0.727323209	0.007348913
Tyrosine	Igsf10	0.530726324	0.075847588
Tyrosine	Incenp	-0.640700426	0.024783535
Tyrosine	Itih1	-0.660544328	0.019378043
Tyrosine	Klra5	-0.728927242	0.007156562
Tyrosine	Lipg	0.780139762	0.002759582
Tyrosine	LOC102546966	0.748623135	0.005089875
Tyrosine	LOC102549836	-0.767356427	0.003576446
Tyrosine	Matk	-0.817419681	0.001165674
Tyrosine	Mlana	0.807866356	0.001478713
Tyrosine	Mnda	-0.653244401	0.021254584
Tyrosine	Mogat2	-0.750454476	0.004923712
Tyrosine	Morn5	-0.776411332	0.00298136
Tyrosine	Mttp	-0.717772134	0.008575631
Tyrosine	Phospho1	-0.78265691	0.002617123
Tyrosine	Pik3c2g	-0.732246912	0.006770494
Tyrosine	Plin2	-0.798121854	0.001860778
Tyrosine	Prc1	-0.507421258	0.092189793
Tyrosine	Ptchd3	-0.805739152	0.001556441
Tyrosine	S1pr4	-0.73756297	0.006185094
Tyrosine	Sult1c3	-0.792062902	0.002133809
Tyrosine	Tf	-0.873122963	0.000208471
